# Preferences and Listening Efficiency of Adults With Cochlear Implants During Online Communication

**DOI:** 10.1097/AUD.0000000000001702

**Published:** 2025-09-04

**Authors:** Francisca Perea Pérez, Douglas E. H. Hartley, Pádraig T. Kitterick, Ian M. Wiggins

**Affiliations:** 1 School of Psychology, University of Nottingham, Nottingham, United Kingdom; 2 National Institute for Health and Care Research (NIHR) Nottingham Biomedical Research Centre, Nottingham, United Kingdom; 3 Hearing Sciences, Mental Health and Clinical Neurosciences, School of Medicine, University of Nottingham, Nottingham, United Kingdom; 4 Nottingham University Hospitals NHS Trust, Nottingham, United Kingdom; 5 National Acoustic Laboratories, Sydney, New South Wales, Australia.

**Keywords:** Captions, Cochlear implants, Decision-making model, Linear ballistic accumulator, Listening efficiency, Online communication, Video calls

## Abstract

**Objectives:**

In recent years, there has been a profound increase in the use of remote online communication as a supplement to, and in many cases a replacement for, in-person interactions. While online communication tools hold potential to improve accessibility, previous studies have suggested that increased reliance on remote communication poses additional challenges for people with hearing loss, including those with a cochlear implant (CI). This study aimed to investigate the preferences and speech-reception performance of adults with a CI during online communication.

**Design:**

An online experiment was designed to study user preferences and listening efficiency during online communication under three presentation modes: “audio” (audio only), “video” (audio-visual), and “captions” (audio-visual plus captions). Fifty adults with at least 1 CI and 50 approximately age-matched people with acoustic hearing (AH) who did not use any hearing devices participated in the experiment. While viewing examples of prerecorded online conversations, participants were asked to indicate which presentation mode they would have preferred had they been participating in those online conversations themselves, and to explain why. Subsequently, participants performed a behavioral test of speech recognition using the same three presentation modes. A joint analysis of accuracy and response time was performed using a hierarchical linear ballistic accumulator model of choice decision-making to derive a measure of listening efficiency.

**Results:**

While people with AH preferred traditional audio-visual presentation, most people with a CI preferred the caption presentation mode, reporting that this helped them follow the conversations more easily. However, participants also reported some perceived drawbacks of captions, related to having to attend to an additional stream of information. In the speech-recognition test, people with AH achieved similar performance across all three presentation modes, while for people with a CI listening efficiency was enhanced by the addition of visual cues: relative to audio-only presentation, people with a CI benefited from the addition of video (i.e., being able to see the talker’s face), and benefited further from the addition of captions. However, even with captions, listening efficiency for people with a CI remained below that achieved by people with AH.

**Conclusions:**

Captions to online conversations are valued by, and beneficial to, people with a CI, but are not without some drawbacks. Lower listening efficiency for people with a CI, even when captions are enabled, may be one reason behind reports in the literature that people with a CI often find online communication to be a cognitively effortful task. Further research is warranted into solutions to help close the performance gap that exists between people with a CI and their AH peers during online communication.

## INTRODUCTION

Accelerated by the coronavirus disease 2019 (COVID-19) pandemic, there has been a profound increase in the use of remote online communication as a supplement to, and in many cases a replacement for, in-person interactions in recent years. During the pandemic, a so-called “new normal” emerged where social and working life, as well as access to essential services, took place almost exclusively online. The transition toward online-delivered services reached almost all sectors of society, including healthcare services (Bokolo 2020; Reay et al. 2020; White et al. 2021), education (Hyseni Duraku & Hoxha 2020), retail (Bhatti et al. 2020), and remote working (Phillips 2020; Kniffin et al. 2021; Wang et al. 2021).

Online communication is sometimes challenging since technical difficulties frequently arise: network-related limitations in speech quality (Markopoulou et al. 2003), stuttering video streams, and lags between audio and video streams are some of the commonly occurring problems. Such disruptions could impede effective communication for everyone, but especially for people with hearing loss (HL) (Kozma-Spytek et al. 2019). In fact, remote communication is considered a significant challenge for people with HL. Previous studies showed that the increased reliance on remote communication during the pandemic imposed an additional burden on people with HL (Ideas for Ears Ltd 2020; Naylor et al. 2020; Tavanai et al. 2021; Saunders & Oliver 2022).

Among people with HL, those with a cochlear implant (CI) might be expected to be particularly badly affected since they have a higher underlying degree of HL (i.e., severe-to-profound HL) and the degree to which a CI can restore hearing to “normal” is inherently limited due to persisting distortions in the frequency, temporal, and amplitude domains (Macherey & Carlyon 2014). A recent study showed that, among adults with HL, people with a CI experienced greater challenges in communication and healthcare access during the COVID-19 pandemic (Wilson et al. 2021). Naylor et al. (2020) similarly showed that participants with greater HL generally reported inferior performance during online video calls compared with in-person conversations. Likewise, an online survey that we conducted previously (Perea Pérez et al. 2022) showed that people with a CI reported high levels of listening effort, among other difficulties, during telephone and video calls, which led them to avoid both modes of remote communication at times. Overcoming such difficulties was important during the COVID-19 pandemic to stay connected to others and mitigate the psychological effects associated with sustained periods of isolation (WHO 2020). Nonetheless, effective virtual communication continues to be important in post-COVID society where online communication remains prevalent (Kane et al. 2021; Masalimova et al. 2021).

Multiple organizations attuned to the difficulties that people with HL faced during the pandemic published advice for effective communication during virtual meetings (ASHA 2020; NDC 2020; Reed et al. 2020; UCL 2020; Maru et al. 2021; RNID 2022). These recommendations covered different aspects of the communication experience such as the speaker’s behavior (e.g., facing the camera, speaking slowly and in turns, rephrasing rather than repeating information), environmental factors (e.g., reduced background activity, adequate lighting), and technological solutions (e.g., video cameras turned on; use of live transcriptions, headphones, and close microphones; recording of meetings; direct streaming of audio to hearing devices).

Regarding the technological solutions, steps taken to improve the quality with which speakers’ voices are captured and to minimize acoustic background noise are highly likely to be beneficial, since speech quality and, especially, signal to noise ratio are well-known factors influencing speech intelligibility (Whitmer et al. 2016; Arehart et al. 2022). Similarly, ensuring that video cameras are turned on and that the speaker’s face is well-lit is likely to be important, since previous research has already demonstrated that people with a CI especially rely on congruous auditory and visual information to optimize communication performance (Moberly et al. 2020). People with normal hearing (NH), or lesser degrees of HL, can also benefit from access to visual cues, especially under challenging listening conditions (Bernstein et al. 2004).

Whether live transcriptions are consistently beneficial to people with HL is, however, not so clearly established. Previous research has found that captioning helps to improve the understanding of televised content by students who are deaf and people who wear a hearing aid (HA) (Jelinek Lewis & Jackson 2001; Gernsbacher 2015; Sharma & Raghunath Rao 2017). However, other studies found that captions do not significantly change the overall level of information assimilation of individuals with HL (Gulliver & Ghinea 2003). Instead, Gulliver and Ghinea’s (2003) study concluded that captions caused a shift in attention from video information to caption information. The need to frequently shift the focus of attention may be one reason why assimilating concurrent audio, visual, and textual information can be a difficult task (Ghinea & Thomas 1998). A study by Jensema et al. (2000) that investigated the eye movements of six people with NH while watching video segments with and without captions confirmed that the addition of captions resulted in major changes in eye-movement patterns, with the viewing process becoming primarily a reading process. Nonetheless, they noticed individual differences, with people accustomed to lipreading spending more time looking at the actors’ lips. In short, it is unclear to what degree people with a CI benefit from having captions available to support speech comprehension during online communication, given a potential increase in cognitive load associated with rapid attentional shifting between video and captions. Further challenges might arise when having to integrate audio-visual information with live (and oftentimes imperfect) captions, although recent developments in AI-powered captioning systems are resulting in rapid improvements in the accuracy of automatically generated captions (Romero-Fresco & Fresno 2023).

Videoconferencing itself is a tiring task for many people. Indeed, the term “Zoom fatigue” was coined in March 2020 to describe the feelings of being overly drained after a period of meeting over a videoconference tool (Bennett et al. 2021). One possible explanation put forward for this phenomenon was that during video calls more attention is paid to nonverbal cues like facial expressions, the tone and pitch of the voice, and body language, consuming mental energy. People with a CI, being already susceptible to experiencing elevated levels of listening effort and listening-related fatigue in everyday life (Alhanbali et al. 2017; Perea Pérez et al. 2022; Philips et al. 2023), may be at heightened risk of experiencing “videoconferencing fatigue” if online communication is more cognitively demanding for them compared with their peers with NH.

The aim of the present study was to provide evidence, subjective and objective, concerning the benefits, or otherwise, of key videoconferencing features for people with a CI. In pursuit of ecological validity, we felt it was important to take this research outside of the laboratory and into people’s homes, to be closer to participants’ true lived experiences with online communication platforms. To that end, we designed an online experiment with novel stimuli and tasks to investigate user preferences and listening efficiency during online communication under three presentation modes: “audio” (audio only), “video” (audio-visual), and “captions” (audio-visual plus captions). The study aimed to examine first which presentation mode was preferred by people with a CI, compared with people with acoustic hearing (AH), when attending to examples of prerecorded online conversations. We wanted to know whether most people with a CI would choose to have captions enabled during video calls, and, if so, what the perceived benefits were (and drawbacks, if any). Subsequently, participants’ speech-recognition performance was measured in an objective behavioral task. We quantified performance using a listening efficiency metric, a type of measure that jointly analyses accuracy and response time to provide insight into the underlying efficiency of auditory cognitive processing (Prodi et al. 2010; Perea Pérez et al. 2023). This metric has been shown to reflect both intelligibility and listening effort, responding to small changes in task demands (Perea Pérez et al. 2023), and we, therefore, postulated that it may be sensitive to differences in cognitive load during online communication. In short, an efficient listener is one who can arrive at a correct understanding of what was said, quickly. We were interested in knowing whether the availability of visual cues and captions in an online context could objectively improve listening efficiency for people with a CI, and, if so, whether they would be enough to close the performance gap between people with a CI and people with AH.

## MATERIALS AND METHODS

The study received ethical approval from the North West—Greater Manchester Central Research Ethics Committee (REC reference: 20/NW/0141).

### Participants

Two groups of participants were recruited: a group of adults with at least one CI and a group of approximately age-matched people with AH who did not use a hearing device in either ear. All participants met the following inclusion criteria (by self-report): aged 18 or over; fluent English speaker; normal or corrected-to-normal vision; capable of giving informed consent, with no known cognitive impairments (e.g., dementia); able and willing to complete the study. In addition, participants in the CI group were required to have at least one CI, while those in the AH group confirmed that they had no worse than mild-to-moderate self-perceived HL and that they did not use any hearing devices. The experiment took approximately 30 min to complete, and participants received a £5 gift voucher.

Full details of participant demographics and hearing profiles are provided in Results, but, in brief, participants in the CI group were experienced users (≥2 yrs postimplantation) with varying age at onset of deafness and a mixture of hearing device configurations (unilateral/bimodal/bilateral). Most participants in the AH group self-reported having no HL in either ear, although a sizeable minority (30%) self-reported slight losses in one or both ears. In the absence of audiometric data, we are unable to say what proportion of participants in this group would have met conventional clinical criteria for NH, hence our use of the generic “acoustic hearing” label for this group.

### Distribution

The online experiment was open for participation from October 8, 2021, until January 7, 2022. People with a CI were recruited first, primarily through the NIHR Nottingham BRC Hearing Theme participant database. An invitation e-mail containing the link to the experiment was sent to eligible candidates. The test was also distributed by national and regional hearing charities and organizations in the United Kingdom including the Royal National Institute for Deaf People (https://rnid.org.uk/), the National Cochlear Implant Users’ Association (https://www.nciua.org.uk/), the British Association of Teachers of the Deaf (https://batod.org.uk/), and Hearing Link (https://hearinglink.org/).

Once the target recruitment figure had been met for the CI group, recruitment of age-matched participants with AH began. These participants were also recruited through our local participant database and other national charities and organizations, such as Age UK (https://ageuk.org.uk), the University of the Third Age (https://u3a.org.uk/), and Life Cycle UK (https://lifecycleuk.org.uk). The experiment was also advertised locally within the Faculty of Medicine and Health Sciences at the University of Nottingham.

### Procedure

The online experiment was implemented using Labvanced (www.labvanced.com), a web-based platform for online experimentation (Finger et al. 2017). It consisted of three main sections: (1) demographic (age, gender, employment) and hearing information (HL severity, hearing-device use, and methods of communication in daily life) questionnaire; (2) video-call preference task; and (3) behavioral speech-recognition task.

Participants were required to answer all questions, although some items (conditional questions) in the demographic and hearing information questionnaire were only displayed when relevant (e.g., only participants who used a CI were asked about their number of years of experience using the device). For a full reproduction of the demographic and hearing questionnaire items, see Supplemental Digital Content 1, https://links.lww.com/EANDH/B687.

After the questionnaire, some preliminary setup instructions were provided to ensure a suitable reproduction of the experiment on each individual’s computer or tablet. Participants with a CI were instructed to use their hearing devices (CIs and, where relevant, contralateral HA) as they would normally do in daily life. All participants were asked to adjust the volume of their computer or tablet to a comfortable level (not too quiet, not too loud) while an example stimulus was played. They were also asked to indicate which sound reproduction (computer/tablet loudspeakers, headphones, direct input/streaming to CI device, other), and user input method (touchscreen device, computer with mouse) they were using. Participants were instructed to use the methods and settings that they would normally use during a video call. Participants were requested to use the same method and settings throughout the experiment. Although this approach to online experimentation cannot control for differences in sound quality and absolute presentation level across participants, it does promote presentation conditions that are ecologically valid, helping to make results more applicable to participants’ real online listening experiences.

**Fig. 1.**
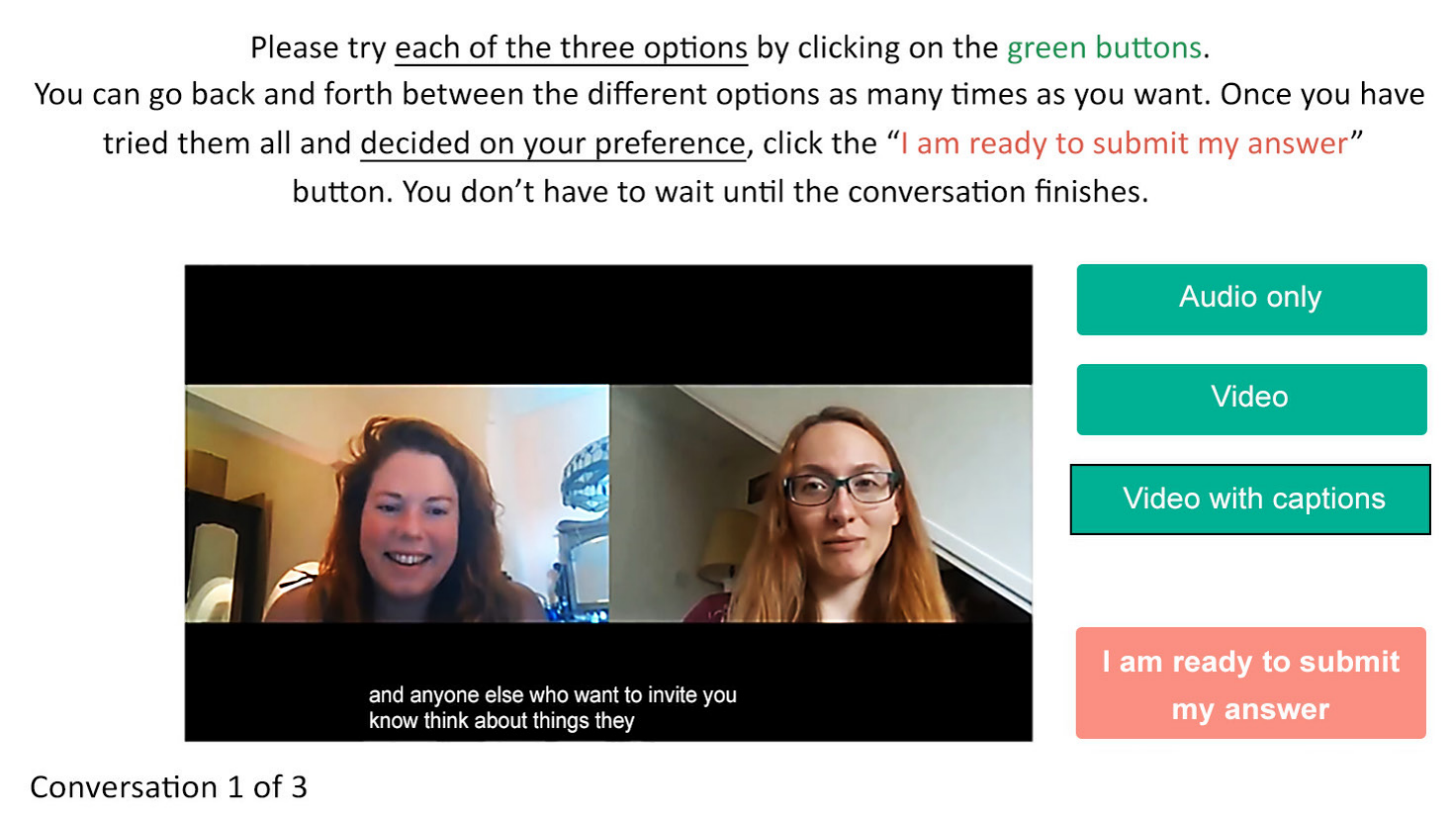
Screenshot of the video-call preference task (conversation B, “captions” presentation mode).

Participants then accessed the second section (preference task) that was designed to collect their preferences and opinions about different video-call presentation modes. Three examples of prerecorded online conversations were presented to participants in random order, each of which was displayed in the three different presentation modes (“audio,” “video,” and “captions”). Figure 1 shows a still from one of the prerecorded online conversations in the “captions” presentation mode. Participants were required to try each mode, switching at will between them, before deciding on their preference (“Which presentation mode would you prefer to use if this was a real video call that you were involved in?”). There were no time restrictions, and therefore participants could spend as much time as they wanted viewing each mode. The conversation recordings (6 min in duration) were repeated in a loop until a decision was made. Once participants had tried each mode and were ready to submit their preference, two additional free-text questions were presented: (i) “Please tell us why this was your preferred mode,” and (ii) “Were there any downsides to your preferred mode?”

Subsequently, participants performed the behavioral speech-recognition task under the same three presentation modes. The task comprised 60 trials in total, 20 per presentation mode, delivered in random order.

**Fig. 2.**
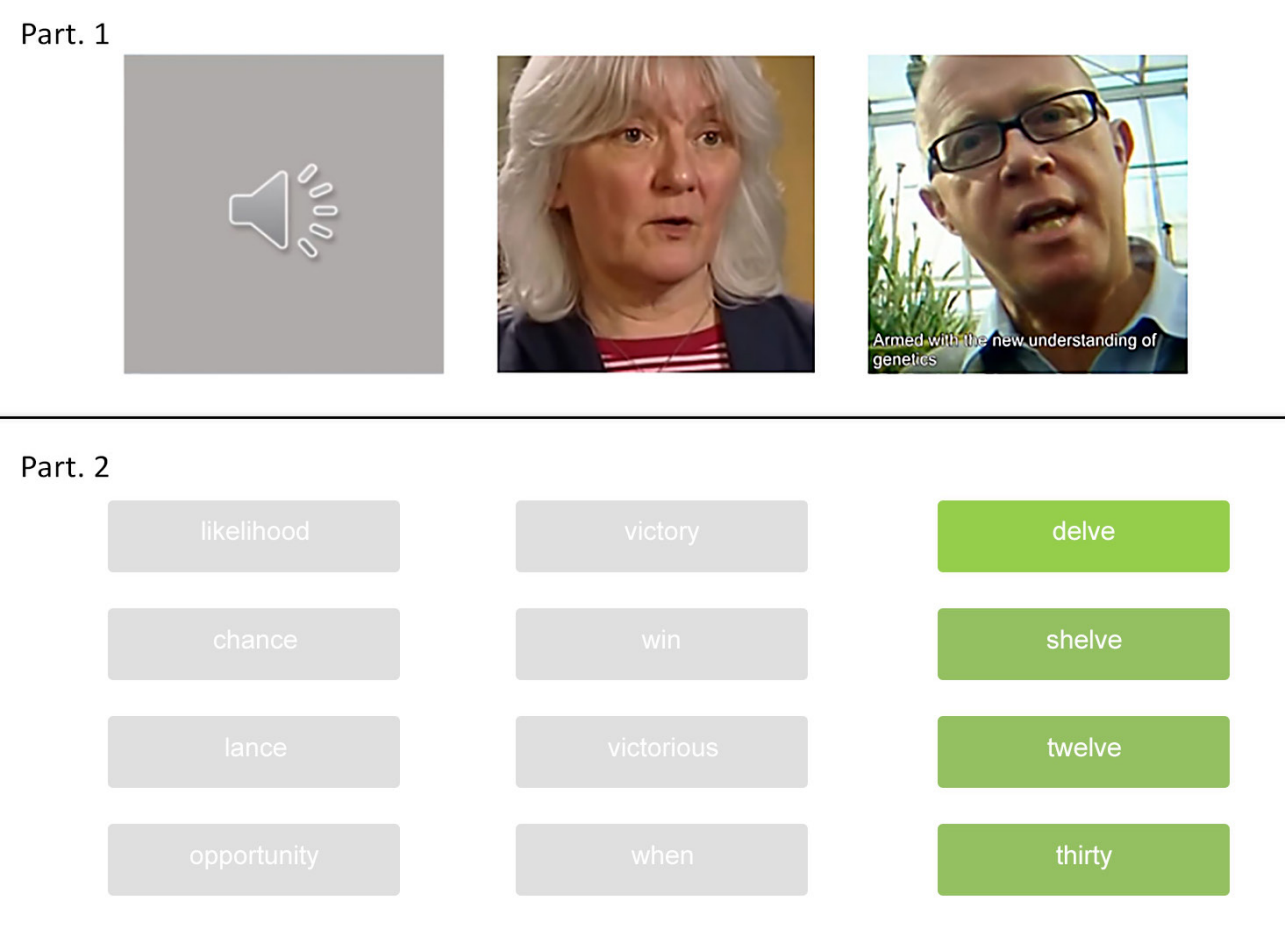
Schematic representation of a trial in the speech-recognition task. First, a sentence-length clip from the LRS2-BBC database was presented under one of the “audio,” “video,” or “captions” presentation modes. Clips were presented full screen for captions to be easily readable when present. After the presentation of the clip, participants were required to sequentially select the correct keywords from closed sets of options. In the example shown (sentence: “when Lorna will have the chance to win up to twelve”), the first two keywords have already been selected, and the third keyword “twelve” is to be selected from among its alternatives (“delve,” “shelve,” and “thirty”).

Each trial comprised a sentence-length clip, of average duration 2.5 sec (SD: 0.2 sec), immediately followed by capture of a participant’s responses (Fig. 2). Depending on the experimental condition, the clips were displayed either as an audio (no visual information, loudspeaker icon shown on screen) or a video clip (with/without captions). Although the same video clips were presented to all participants, each participant received a different (pseudorandom) allocation of the clips to the three presentation modes. Playback began automatically at the start of each trial, and no repetition was possible.

After presentation of the clip, the participant was required to select three keywords that had appeared in the clip. Each keyword was presented in turn alongside three alternative response options, one of which was semantically similar to the keyword, one of which was phonetically similar, and one of which was dissimilar. These four words (the keyword and the three alternative words) were displayed on the screen as green buttons positioned along a vertical column (in random order). Once a response was given, the next set of four words (the next keyword and its alternatives) appeared on the screen. When a new set of words appeared, the previous sets remained visible, although in dimmed gray color to indicate that the buttons were no longer active. Response time was recorded for each keyword selection from the moment in which the set of words appeared on the screen until the moment that one word of the set was selected. There were no time restrictions, so participants could take as much time as they needed to select the words. However, participants were instructed to give equal weight to speed and accuracy when making their responses, and to give their best guess if unsure. As soon as a word from the final set of a given trial had been selected, the next trial started automatically, after a short delay. Participants were permitted to take a break mid-test, after having completed 30 trials. Before the task, participants completed a short practice session with six trials, two per presentation mode, to become familiar with the task (the results from which were discarded).

Participants were not given any information concerning the accuracy of the captions presented during each section of the experiment, which were perfectly accurate on the speech-recognition task, but only moderately accurate on the preference task (refer to details of stimulus generation in Materials and Stimuli).

### Materials and Stimuli

#### Video-Call Preference Task

The three example conversations used in the preference task were recorded using Microsoft Teams and Zoom platforms. Six adult volunteers, 5 native English speakers, and 1 non-native English speaker, from the local research group consented to the audio-visual recordings being used as stimuli in the present study. To simulate natural video calls, the conversations were unscripted. Nonetheless, each conversational couple were given a topic as a starting prompt, from which the conversation could develop freely. Speakers were instructed to carry on with the conversation in the same way that they would normally do during a video call, even if any connection problems or technical difficulties arose. Details of the three conversations were as follows:

- Conversation A. Speakers: female (native English speaker) and male (non-native English speaker). Topic: “Discuss which three personality traits/qualities you both like (admire) and dislike in a person and why.” Platform: conversation recorded using Microsoft Teams with a video frame rate of 8 frames per second (fps) and an audio sampling rate of 16 kHz. Quality: there were some connection problems and/or perceived difficulties including poor audio and video quality due to the low recording frame rate, poor lighting which partially dimmed one speaker’s face, and the presence of some overlapping speech during the conversation.
- Conversation B. Speakers: 2 females (both native English speakers). Topic: “Discuss and plan a dinner party that you would have together using only food and drink you both dislike.” Platform: conversation recorded using Zoom, with a video frame rate of 25 fps and an audio sample rate of 32 kHz. Quality: the camera image for one of the speakers was slightly blurred, otherwise there were no obvious audio or video quality issues.
- Conversation C. Speakers: 2 females (both native English speakers). Topic: “You both have won an all-inclusive holiday together (all expenses paid). Discuss and agree on where you want to travel to, the type of accommodation, for how long you will be there, and the activities you will book.” Platform: conversation recorded using Zoom, with a video frame rate of 25 fps and an audio sample rate of 32 kHz. Quality: no connection problems or perceived difficulties were apparent.

These conversations were selected as stimuli because they illustrated a range of different video-call qualities that could naturally occur in real life, though the quality of the recordings was not intentionally manipulated. Captions for the three conversations were generated using YouTube (Google LLC) automatic captioning in June 2021, which generated versions of the original videos with captions embedded for use in the relevant conditions of the experiment. Captions appeared in a non-serif white font over black background, at the bottom of the video frame, with up to two lines of text shown at a time. Words appeared sequentially as the conversation unfolded, in the style of live (real-time) captioning. The captions did not convey any information about which conversational partner was speaking at any given moment.

#### Speech-Recognition Task

Sixty-six video clips from the Oxford-British Broadcasting Corporation Lip Reading Sentences 2 (BBC-LRS2) Dataset were used as stimuli in the speech-recognition task (60 in the main task and 6 in the practice session). This database has previously been used in research for audio-visual speech recognition (Chung et al. 2017; Son & Zisserman 2017; Afouras et al. 2019). It consists of several thousand spoken English sentences, covering a wide range of topics, taken from various BBC television programmes. Facial recognition analysis was carried out using OpenFaceR toolkit as described by Cannata and Redfern (2020), to select only those videos that were, to a first approximation, comparable to typical video-call conversations. Parameters describing head position, gaze, and head rotation movements were limited to a range around zero, to identify videos in which the speaker’s head was in a central position, gaze toward the camera, with limited head movements. Video clips were further filtered based on other parameters of interest including duration (between 2 and 3 sec), video quality (≥1.5 Mb/s), and word count (≥six words). In total, 355 video clips met the full set of criteria and were selected for use in the behavioral task.

The transcriptions provided with the BBC-LRS2 videos were used to generate response options for the behavioral task. First, we identified candidate keywords within sentences using “spaCy”’ (https://spacy.io/), a Python-based open-source natural language processing package that classifies words according to their syntax. Candidate keywords were nouns, verbs, adjectives, adverbs, or numerical values. Alternative response options for each candidate keyword were then generated as follows. A list of semantically similar words was generated using the Gensim “Word2Vec” model. Separately, a list of phonetically similar words was generated using the “get_rhymes” function from the Python package “English to IPA” (https://pypi.org/project/eng-to-ipa/), which converts English text into the International Phonetic Alphabet (IPA). For all the generated words across these two lists, phonetic and semantic similarity scores to the original keyword were calculated using the “Metaphone” and “Word2Vec” algorithms, respectively. Checks were run to ensure that all the generated alternative words existed in the English language (using the “Brown Corpus” and the “word2vec-google-news-300” model as dictionaries), and that none of them were identical to the original keyword.

Final selection of keywords and alternatives was done in R (Version 4.1.2; R Core Team, 2021) with manual intervention by author FPP. For each video clip, 12 words were selected in total: 3 keywords plus, for each keyword, a “semantically similar,” a “phonetically similar,” and a “dissimilar” alternative response option. Alternative response options were selected based on semantic and phonetic similarity scores to the relevant keyword. The “ideal” semantically similar alternative was a word with maximum semantic similarity to the keyword and minimum phonetic similarity. Conversely, the “ideal” phonetically similar alternative was a word with minimum semantic similarity and maximum phonetic similarity to the keyword. Finally, the “ideal” dissimilar alternative was a word with minimum semantic and phonetic similarity to the keyword. Manual checks were carried out to ensure that each of the three alternative words were valid and different from one another. A spreadsheet containing 265 video clips was created, including the 12 words needed per video clip for use in the behavioral task.

Captioned versions of the 265 videos were created using a custom MATLAB script to generate scrolling-text captions based on the sentence transcriptions, with the results burned into the media file using FFmpeg (Version 4.4; FFmpeg developers, 2000–2021; http://ffmpeg.org/). Captions appeared in a non-serif white font with black shadow, overlaid towards the bottom of the video frame. Words appeared sequentially as the clip unfolded, in the style of live (real-time) captioning. Ultimately, 66 video clips (20 video clips per presentation mode plus 6 practice clips) were randomly selected from the 265 pre-prepared videos for use in the behavioral task.

### Analysis

Anonymized participant response data were exported from the Labvanced platform as comma-separated values files. Group-level differences regarding video-call preferences were analyzed with Bayesian statistics using the “BRMS” R package (Bürkner 2017). Poisson regression models were fitted to examine the effect of presentation mode and group (formula: Counts ~ Mode × Group), and, in a subsequent three-level model, the effect of presentation mode, group, and specific conversation (formula: Counts ~ Mode × Group × Conversation). Default noninformative prior distributions were used, assuming that all values within plausible ranges were equally likely a priori. Posterior distributions were estimated using the Markov Chain Monte Carlo (MCMC) (Van Ravenzwaaij et al. 2018) algorithm, with convergence confirmed based on the Rhat statistic (Brooks & Gelman 1998). For each model, 4 independent chains were run, each with 2000 warmup iterations followed by another 2000 post-warmup iterations. Posterior predictive checks were performed to ensure that model predictions adequately fitted the data. The model conditional effects, predicted means, and 95% credible intervals were reported per group and condition.

In the speech-recognition task, we quantified performance using the listening efficiency metric described by Perea Pérez et al. (2023). Listening efficiency is an integrative measure of accuracy and response time, with high listening efficiency meaning that a person can quickly arrive at an accurate understanding of what was said; lower listening efficiency means that a person makes more errors and/or takes longer to arrive at a correct understanding. Our approach involves building a hierarchical linear ballistic accumulator (LBA) model to explain participants’ trial-level behavioral responses, which is fit using Bayesian methods and a measure of listening efficiency is derived from the model parameters. The LBA is a simplified computational model of how evidence is accumulated to make decisions (Brown & Heathcote 2008). In the LBA model, evidence in favor of a particular response is assumed to grow linearly over time, with each response option having its own evidence accumulator; the decision-making process is modeled as a race between these competing accumulators, the selected response being the one whose accumulator is first to reach a certain “response threshold.” The average rate at which evidence accumulates over time is termed the “drift rate” and can differ for each response option. The LBA model additionally includes parameters for the maximum amount of “starting evidence” (bias) toward each response option at the start of a trial and for “non-decision time,” which captures the time taken to encode the stimulus and to generate the physical response (i.e., mouse click or tap). The model is hierarchical in the sense that individual participants are modeled as belonging to a higher-level group (CI or AH), and both group-level and individual-level parameters are estimated simultaneously. The estimation of individual-level values for each model parameter is akin to including random intercepts and slopes in a conventional linear mixed model analysis.

The LBA model was implemented in Stan (Stan Development Team 2024) using the CmdStanR interface (Gabry et al. 2025) . Our implementation was based on the code provided by Annis et al. (2017), modified in two important ways: (i) by using a noncentered parameterization to efficiently explore the posterior parameter distributions* (https://mc-stan.org/docs/2_18/stan-users-guide/reparameterization-section.html); and (ii) by using Stan’s “reduce_sum” facility to speed up model fitting through within-chain parallelization (https://mc-stan.org/docs/stan-users-guide/parallelization.html#reduce-sum). To handle the possibility of spurious responses (due, e.g., to occasional lapses of concentration), we mixed the LBA model with a contaminant response-time distribution. Contaminant responses were assumed to be drawn from a uniform distribution with bounds of zero seconds and the maximum observed response time. The overall proportion of contaminant responses was estimated from the data within the Bayesian model fitting process.

We followed the approach described by Gunawan et al. (2020) to handle intercorrelation among LBA model parameters. In this approach, LBA parameters at the individual-subject level are modeled as being drawn from a multivariate normal distribution, with the group-level intercorrelation matrix explicitly estimated from the data. Our final model had 17 free parameters per individual: drift rate for evidence accumulation towards each type of response (“correct,” “incorrect–semantic,” “incorrect–phonetic,” and “incorrect–neither”) for each presentation mode (audio, video, and captions); two “drift rate modifier” parameters (vcBoost.Kw2 and vcBoost.Kw3) that were fixed across conditions and which were used to model serial dependency across the keywords of a sentence (e.g., vcBoost.Kw2 described the average increase in the rate of evidence accumulation toward a correct response on keyword 2 if a correct response had been given for keyword 1; vcBoost.Kw3 described the average increase in the rate of evidence accumulation toward a correct response on keyword 3 if correct responses had been given for both keywords 1 and 2); and, finally, parameters *A* (upper value for starting evidence), *b* (response threshold), and *t*0 (non-decision time) which were assumed to take a fixed value across conditions. The model scaling constraint was met by fixing the between-trial variability in drift rates to a value of one. Uninformative priors (normal and Student *t* distributions, as appropriate) were used for all model parameters, with the exception that non-decision time *t*0 was constrained to be above a minimum value of 150 msec. Posterior distributions were estimated using Stan’s default “No-U-Turn-Sampler” MCMC algorithm, over 4 independent chains, each with 1000 warmup iterations followed by another 2000 sample iterations (8000 final draws in total). Posterior predictive checks were used to verify the agreement between model predictions and observed data.

As in our previous study (Perea Pérez et al. 2023), we computed listening efficiency as the differential drift rate toward a correct response: in the present study, listening efficiency equals the drift rate toward a correct response minus the mean of the drift rates toward the three types of error response. In the case of a hypothetical participant making random guesses, the average drift rate toward each type of response would be equal, and so listening efficiency would approach zero. A better-performing participant would accumulate evidence more quickly toward the correct response option than toward any of the error responses, and so listening efficiency would take a positive value, increasing in size as performance improved. Effects were assessed using 95% highest density intervals that act as the 95% credible intervals (CrI) of the mean posterior parameters. We considered the effects of group and/or presentation mode to be reliable when the 95% CrI of the difference between the relevant posterior parameters did not contain zero. For comparison to the LBA-based analysis, separate analyses of accuracy and response time are included as Supplemental Digital Content 4, https://links.lww.com/EANDH/B690.

Open (free-text) questions were analyzed using a simple descriptive approach, with themes and categories selected following Elo and Kyngäs’s (2008) guidelines for inductive content analysis. The resulting themes and categories identified are reported in tables organized by conversation and group (Supplemental Digital Content 2, https://links.lww.com/EANDH/B688).

## RESULTS

### Participant Demographics and Hearing Profiles

One hundred participants completed the experiment, 50 with a CI and 50 with AH. The age of participants in the CI group ranged from 20 to 86 years (M = 64.2, SD = 15.1), with 74% being female. The AH group showed a similar age range (22 to 86 years, M = 59.3, SD = 12.2), with a more balanced gender distribution (54% female). While a majority of the CI group were retired (62%), only 30% of the AH group were retirees. Most AH participants were currently working in part-time (32%) or full-time (30%) jobs.

**Fig. 3.**
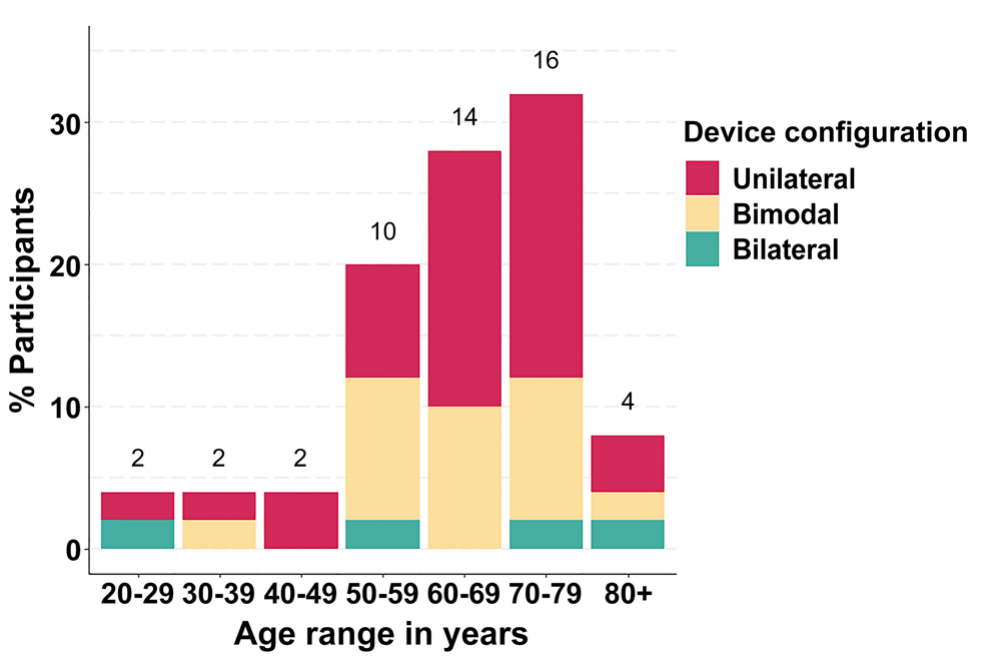
Hearing device configuration by age group for participants with a CI. Number of participants in each age group is shown above each bar. CI indicates cochlear implant.

All participants in the AH group reported having no worse than mild-to-moderate self-perceived HL in either ear and confirmed that they did not use any hearing devices, such as a HA. Nonetheless, 30% of participants in the AH group, mostly aged over 50 years old, reported having some degree of (presumably) age-related HL. Participants in the CI group had on average 10 yrs of experience with implants (range 2 to 26 yrs). The onset of HL in the implanted ear(s) occurred early in life (up to the teens) for 46% of participants, while 52% lost their hearing in middle adulthood (30 to 60 yrs old). Attending to the hearing device configuration, 58% of participants were unilateral CI users (1 CI), 34% were bimodal users (1 CI and a contralateral HA), and 8% were bilateral CI recipients (2 CIs). Figure 3 shows CI users’ hearing device configuration by age group. All participants with one CI reported having severe (6%) or profound (86%) HL in the non-implanted ear. Nearly all bimodal listeners reported using their contralateral HA daily and wearing the same during the experiment.

**Fig. 4.**
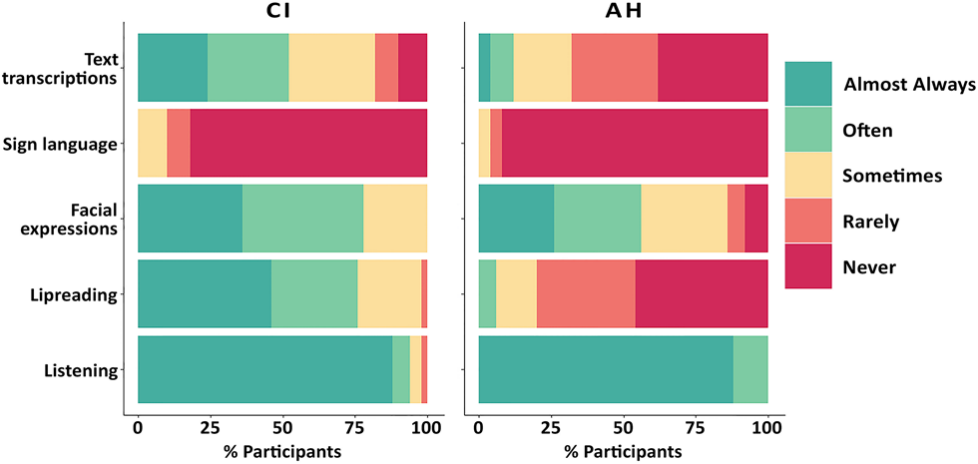
Percentage of participants who rely on different ways of communication (listening, lipreading, facial expressions, sign language, and text transcriptions) in everyday life by group. Q8: “In everyday life, to what extent do you rely on these ways of communication?” AH indicates acoustic hearing; CI, cochlear implant.

Despite marked differences in hearing profile between groups, common features can be seen in Figure 4 regarding ways of communication in everyday life. Nearly all participants (88%) across both groups reported relying almost always on auditory speech for communication. Most participants in both groups reported making regular use of facial expressions to support communication, although participants with a CI did so to a greater extent than those with AH (78% in the CI group versus 56% in the AH group). Participants with a CI also made regular use of other visual cues such as lipreading (76%) or text transcriptions (52%) to further support communication. Conversely, very few participants with AH reported using these methods (6% and 12% for lipreading and text transcriptions, respectively). Few participants in either group reported using sign language to communicate with others (4% and 10% of participants used sign language “sometimes” in the AH and CI groups, respectively).

### User Input and Sound Reproduction Methods

Most participants completed the test using a computer with mouse (90% and 74% in the AH and CI groups, respectively). The remainder used a touchscreen device (e.g., tablet). Instructed to use the sound reproduction mode that they would normally use during an online video call, most participants chose to use loudspeakers (72% and 68% in the AH and CI groups, respectively). Others used headphones to complete the test (28% and 12% in the AH and CI groups, respectively), while a minority (20%) of CI users streamed audio directly to their hearing device(s).

### Video-Call Preferences

**Fig. 5.**
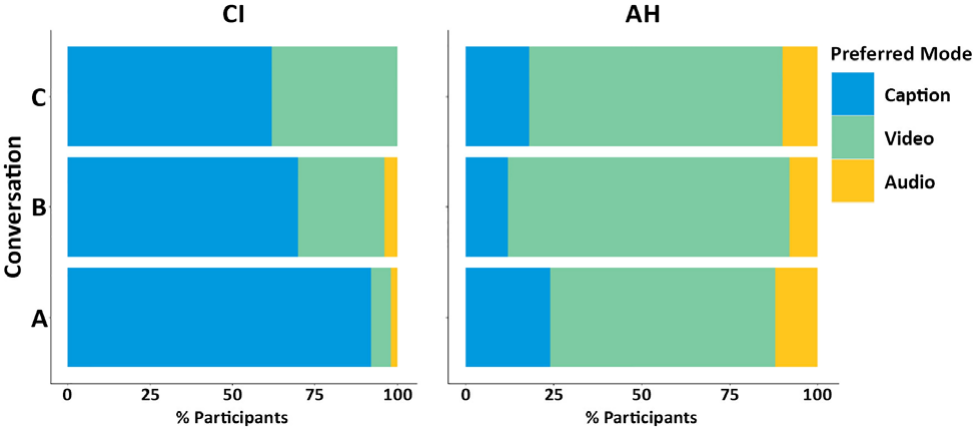
Participants’ preferred video-call presentation mode for each conversation (A, B, C) by group. AH indicates acoustic hearing; CI, cochlear implant.

**Fig. 6.**
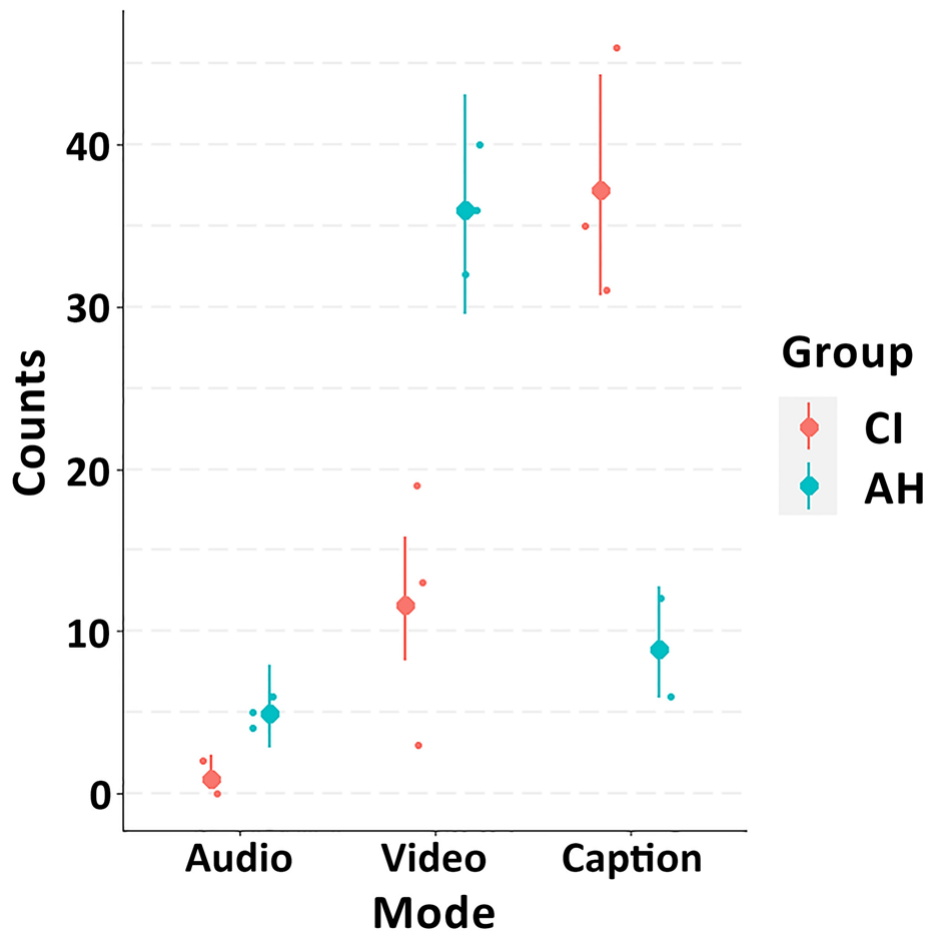
Conditional effects of the Poisson regression model (Counts ~ Mode × Group) plotted over participants’ raw preferences data. The larger circles represent posterior medians, the error bars show 95% credible intervals, and the small circles represent the raw data (counts of preference vote for each presentation mode, by group). AH indicates acoustic hearing; CI, cochlear implant.

As shown in Figure 5, most participants with a CI chose the “caption” presentation mode for all three conversations (92%, 69%, and 63% for conversations A, B, and C, respectively), whereas participants with AH overall preferred the “video” mode (64%, 80%, and 72% for conversations A, B, and C, respectively). These differences were confirmed by the Poisson regression model: non-overlapping 95% CrIs for the conditional effects indicated a robust effect of group on participants’ preferred presentation mode (Fig. 6).

**Fig. 7.**
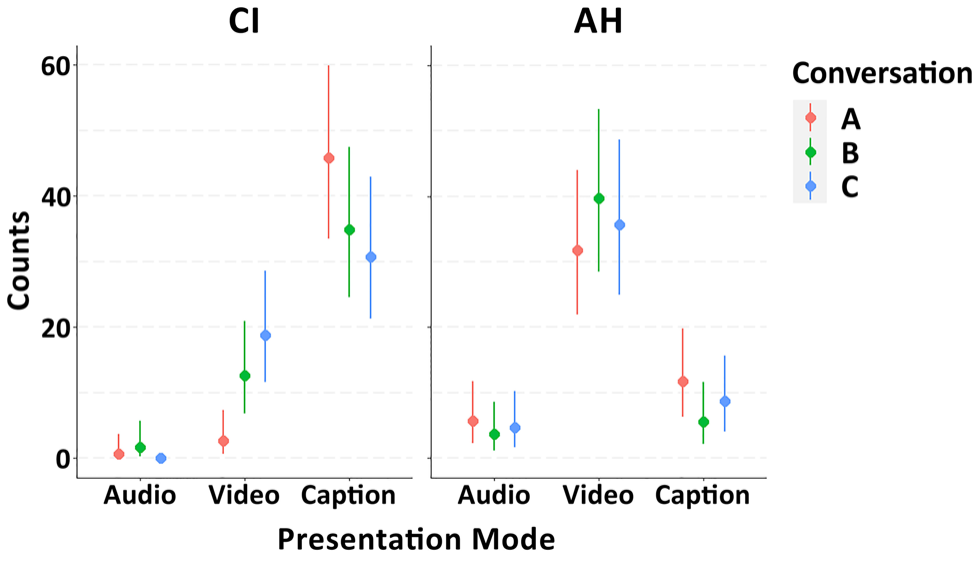
Conditional effects plot of the Poisson regression model for preferred listening mode, including specific conversation as an additional fixed effect (Counts ~ Mode × Group × Conversation). The circles represent posterior medians and error bars display 95% credible intervals. AH indicates acoustic hearing; CI, cochlear implant.

The three-level Poisson regression model that included “conversation” as an additional fixed effect revealed weak evidence for an influence of the specific conversation on preferred presentation mode (Fig. 7). Especially for participants with a CI, the preferred presentation mode seemed to switch increasingly from “video” toward “captions” as the technical (audio-visual) quality of the simulated video call became worse (Conversation C -> Conversation B -> Conversation A). However, the effect of conversation quality was small relative to measurement uncertainty, as reflected by overlapping 95% CrIs.

### Listening Efficiency

A total of 18,000 observations (60 trials × 3 keywords per sentence × 100 participants) were included in the LBA model. No outlier removal was performed since our model included a built-in contaminant response-time distribution which would naturally account for unexpectedly fast or slow responses. Satisfactory convergence was confirmed for all estimated parameters through visual inspection of the MCMC chains and by Rhat values ≤1.01 (Rhat = 1 at full convergence). Posterior predictive checks confirmed that model-generated response-time distributions closely followed the observed data, and modeled accuracy was within 1.8%-points of observed accuracy for all combinations of group and presentation mode (Supplemental Digital Content 3, https://links.lww.com/EANDH/B689).

**Fig. 8.**
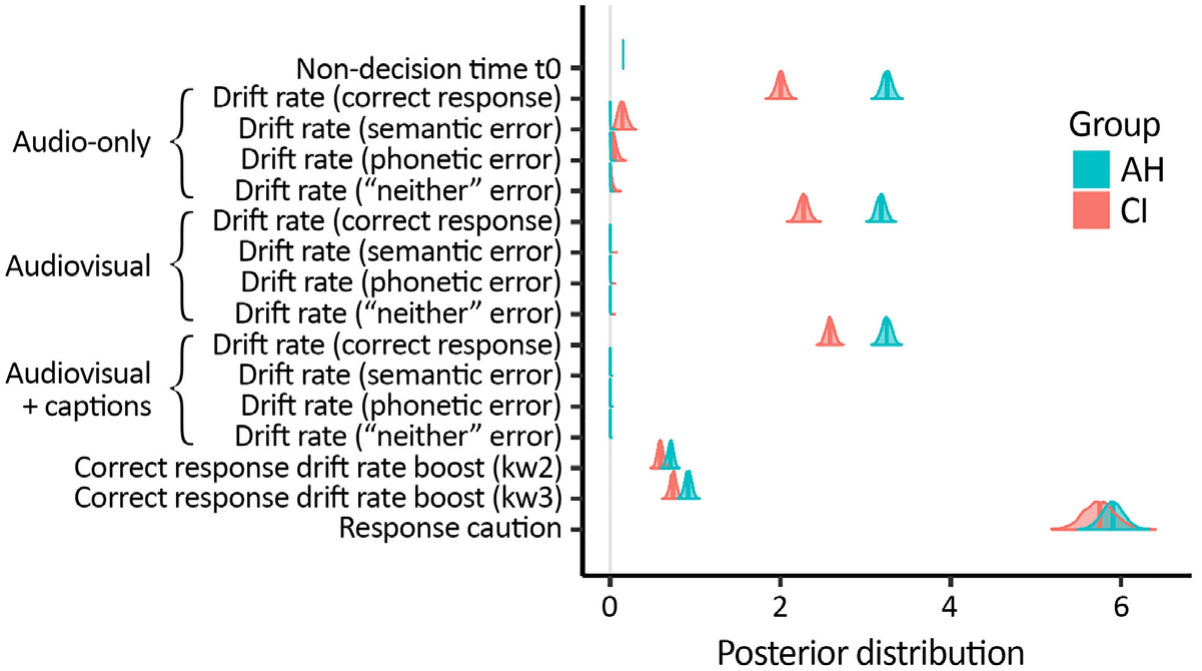
Posterior distributions for group-level LBA model parameters. Parameters are (from top to bottom): non-decision time (t0); evidence accumulation drift rates for each response type (correct, semantic-error, phonetic-error, “neither”-error), grouped by presentation mode (audio, video, captions); two correct response drift-rate boosts (for keyword 2 and keyword 3, respectively) to model serial dependency across keywords within a sentence; and response caution. Solid vertical lines represent median values and the distributions extend to cover the central 99% probability mass of the posterior. AH indicates acoustic hearing; CI, cochlear implant.

Figure 8 shows the posterior distributions for the group-level LBA model parameters. Addressing first the commonalities between groups, there was no evidence for a group difference in either non-decision time (*t*0) or response caution†. This indicates that any differences in performance between the CI and AH groups were not due to, for example, one group being slower than the other to perform their motor responses, or one group adopting a more cautious approach to the speed-accuracy trade-off overall; rather we can attribute any group differences to a difference in the efficiency of auditory decision making. Both groups benefitted from having correctly recognized preceding keywords within a sentence, as confirmed by posterior distributions for the two serial-dependency “boost” parameters that lay well above zero. However, AH listeners appear to have benefitted slightly more (by ~20%) from this within-sentence context effect than people with a CI (credibility of a between-group difference ≥99%).

A very low frequency of incorrect responses overall (0.8% and 12.6% in the AH and CI groups, respectively) precluded an in-depth analysis of error types. However, in the case of participants with a CI listening to audio-only stimuli (for which error responses were slightly more common, occurring at a rate of around 20%), there was some evidence from the relevant drift-rate parameters that, of the three types of error, evidence accumulated most quickly towards words that were semantically similar to the original keyword, followed by phonetically similar words, and with the slowest rate of evidence accumulation towards words that were neither semantically nor phonetically similar to the original keyword.

**Fig. 9.**
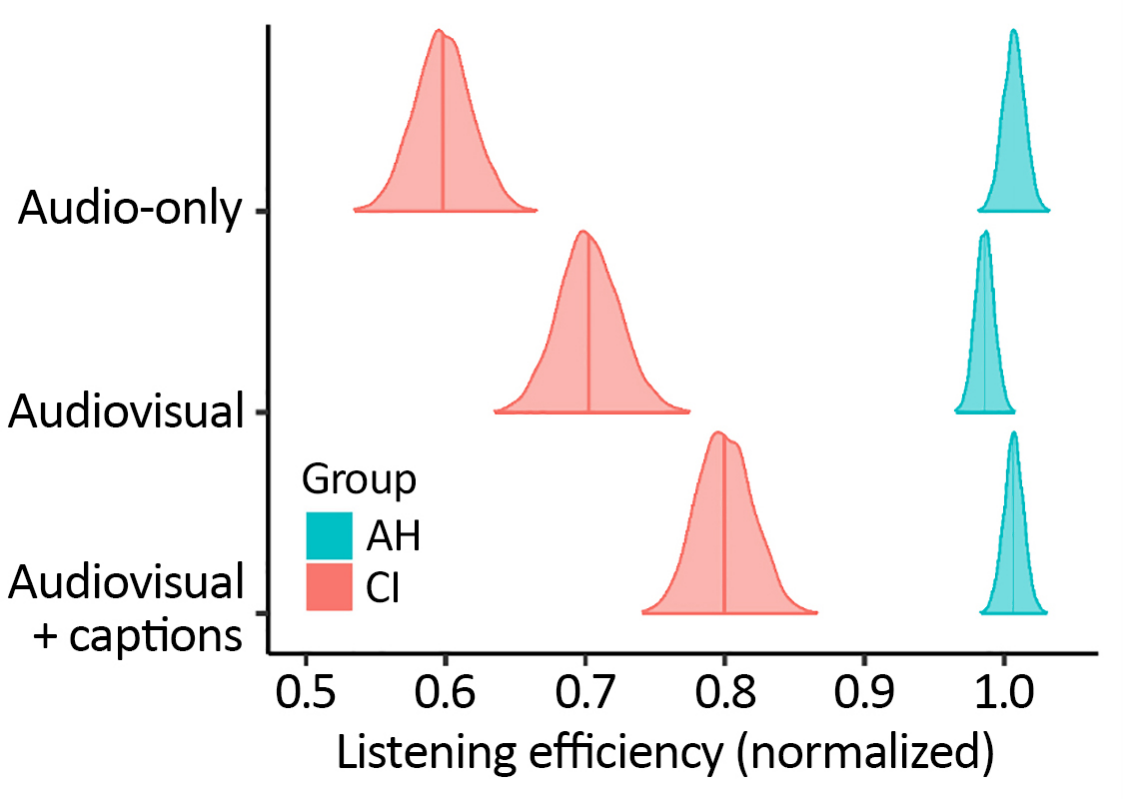
Posterior distributions for derived group-level listening efficiency scores. Scores were normalized such that mean performance for AH listeners (across presentation modes) took a value of one. Vertical lines represent median values and the distributions extend to cover the central 99% probability mass of the posterior. AH indicates acoustic hearing; CI, cochlear implant.

It is clear from Figure 8 that there were substantial between-group differences in drift rates for correct responses. To explore these differences in a more intuitive format, we derived listening efficiency scores from the drift-rate parameters (as described in Materials and Methods) and furthermore, we normalized these scores such that mean performance for AH listeners (across presentation modes) took a value of one (Fig. 9). Two things are immediately evident from Figure 9: first, listening efficiency was overall much lower for people with a CI compared with people with AH; second, for the CI group, listening efficiency varied substantially across presentation modes, whereas for the AH group, listening efficiency was similar regardless of presentation mode. For participants with a CI, listening efficiency was worst for audio-only presentation (~60% of mean listening efficiency for AH participants), improved by the addition of visual information (to ~70% of mean listening efficiency for AH participants), and further improved by the addition of captions (to ~80% of mean listening efficiency for AH participants). We can have confidence in the presence of these differences between presentation modes (within the CI group), owing to high credibility of the associated effects: credibility of higher listening efficiency for (a) audio-visual compared with audio-only presentation and (b) audio-visual presentation with captions compared with audio-visual presentation without captions both ≥99%.

## DISCUSSION

This online study compared the subjective preferences and listening efficiency of a group of 50 people with a CI and a group of fifty controls with AH when attending to prerecorded online video-call conversations and sentence-length video clips, respectively, under three presentation modes: audio, video (audio-visual), and captions (audio-visual plus captions).

### Captions: A Beneficial, But Imperfect, Solution for People With a CI

People with a CI expressed a strong preference for the “captions” presentation mode when listening to prerecorded online conversations, especially in the case of a video call with poorer audio-visual quality, for which nearly all (46 of 50) CI users preferred to have captions enabled. Responses to the associated free-text questions elucidated the reasons behind these preferences. The most prominent themes among CI users’ responses were that captions helped to reduce listening effort and provided increased confidence/confirmation of what was heard, with these benefits being especially important under more challenging listening conditions. Several participants highlighted, however, that accurate and synchronized captions were a prerequisite for these benefits to be realized, with at least one participant reporting that, although they would ordinarily use captions, they selected the “video” presentation mode because they found the captions to be out-of-sync with the speech at times.

Participants both with and without a CI confirmed what previous research by Jensema et al. (2000) has shown: that in the presence of captions people found themselves shifting attention from the visual content (the speakers’ faces) to the text content (captions). Some participants reported a feeling of cognitive effort, or even overload, associated with this need for rapid attention switching, and multiple participants noted that they missed out on some non-verbal cues and facial expressions when attending to the captions. Thus, while captions were generally beneficial to (and favored by) people with a CI, they also had some drawbacks. It seems that, depending on the circumstances, individual characteristics, and intent, captions may alleviate some aspects of listening effort, yet increase cognitive load in other ways. This might go some way toward explaining why, even with technological support, people with a CI report online communication to be a cognitively effortful task (Perea Pérez et al. 2022). In the present study, people with AH could avoid such trade-offs, since they were typically able to understand the conversations without the need for captions.

The speech-recognition task provided complementary objective evidence that visual cues are beneficial in supporting people with a CI to recognize speech more quickly and accurately during online communication. Compared with audio-only performance, listening efficiency for people with a CI was improved by ~17% through the addition of video (i.e., by being able to see the speaker’s face), and improved by a further ~14% through the addition of captions. We would expect these improvements in listening efficiency to reduce the perceived cognitive burden of online communication and help people with a CI to keep up with the pace of online conversations. Nevertheless, even under the most favorable conditions of the present study (reasonably clear speech, with lipreading possible, and accurate captions available), group-average listening efficiency for people with a CI remained ~20% lower than people with AH were able to achieve with audio-only presentation. Thus, the addition of captions during online communication was not enough to close the performance gap that existed between people with a CI and people with AH. It is unclear from the present study to what extent the lingering performance gap reflects a fundamental limit on the speed with which participants with a CI could read and process the captions (had there been no concurrent audio-visual stimulus), or whether listening efficiency was partially compromised by the cost of dividing/switching attention between the audio-visual and textual information streams.

Our participants made some insightful suggestions about how video-call captioning could be improved. Aside from ensuring that captions are as accurate and synchronized with the audio-visual stream as possible, it was suggested that the use of different colored captions to denote who was speaking at any given moment could be helpful, as could presenting the captions closer to the relevant speaker’s face, for example, in a comic-book style. Similar ideas have been the topic of published research (Vy & Fels 2009; Amin et al. 2022). Our participants indicated that even small changes in how captions are displayed, such as using larger fonts and making greater use of relevant punctuation, would make a significant difference in aiding speech understanding while reducing cognitive effort.

### The Importance of Context

The importance of context was evident in the speech-recognition task, in that drift rates toward a correct response in the LBA model were positively boosted when the preceding keyword in the sentence was identified correctly, and boosted still further when the two preceding keywords were identified correctly. These results are consistent with a substantial body of extant evidence that context plays an important role in speech understanding (Eisenberg et al. 2002; Sheldon et al. 2008; Wilson & Dorman 2008; Dingemanse & Goedegebure 2019). Serial dependency for correct keyword understanding within a sentence has similarly been observed in other studies. For instance, Winn and Teece (2020) studied 21 CI users and showed that, in high-context sentences, intelligibility scores for the final words reached 97.8% when preceding words were repeated correctly, whereas the accuracy of final word identification dropped to 56.5% when there was at least one preceding error. It is interesting to note that in our results, the boost in drift rates toward a correct response following correct identification of preceding keywords was greater for AH listeners than for CI users, an effect that has not always been observed in previous research (O’Neill et al. 2021). When participants did make errors in keyword identification (which was rare in the present study, except in the case of people with a CI listening to audio-only sentences), our results indicate that the most common type of error response was to select a word that was semantically similar to the original keyword, more so than one that was phonetically similar. This would seem consistent with Winn and Teece’s (2021) contention that listening may primarily be a search for plausibility and semantic coherence, leading to errors of perception that cannot be predicted by phonetic similarity.

### Advantages of Listening Efficiency as a Measure of Speech-Recognition Performance

The notion of quantifying auditory performance using a measure of listening efficiency was introduced by Prodi et al. (2010) and used in subsequent studies (Prodi & Visentin 2015). A primary motivation for the use of listening efficiency is that it usefully integrates intelligibility (accuracy) and “effort” (as indexed by response time) and may therefore be capable of discriminating between different listener groups or listening conditions even in cases where intelligibility is almost equal. The approach builds on evidence from the wider experimental psychology literature that integrated speed-accuracy scoring is generally more reliable than nonintegrated (separate) scoring of response time and accuracy (Bakun Emesh et al. 2022). This occurs, at least in part, because differences in performance (between individuals and/or test conditions) may variously appear as an effect on accuracy, on response time, or on both, meaning that neither accuracy nor response time alone is guaranteed to capture all the relevant information. Furthermore, on a practical level, using listening efficiency as the performance metric frees a researcher from having to evaluate two separate outcomes, with the concomitant need to consider correction for multiple testing and the possibility of difficulties of interpretation in cases where accuracy and response time lead to contradictory conclusions (Vandierendonck 2017).

In Prodi et al.’s (2010) original formulation, listening efficiency was defined simply as the ratio of an intelligibility score to average response time. Although this approach was shown to have utility, research conducted outside of the auditory field has found that the use of a model-based approach, such as the LBA, to combine accuracy and response time data can provide more sensitive and representative results (Mueller et al. 2020). Furthermore, by providing a principled account of the decision-making process, model-based approaches allow one to discern participants’ underlying abilities unconfounded by speed-accuracy trade-offs or individual differences in “non-decision time,” such as the time taken to execute the motor response (Stafford et al. 2020). Accordingly, in Perea Pérez et al. (2023), we developed a new listening efficiency metric, derived from the differential rate of evidence accumulation toward a correct response in a hierarchical LBA model of choice decision making. We applied that same approach in the present study with the aim of obtaining information about participants’ underlying auditory cognitive processing abilities under different presentation modes.

As discussed in preceding sections, using the LBA-derived listening efficiency metric we were able to identify robust differences in ability between people with a CI and people with AH, as well as robust benefits from the addition of visual cues (including captions) within the CI group. It is interesting to note that, in the present dataset, qualitatively the same conclusions could have been drawn from separate analyses of accuracy and response time (Supplemental Digital Content 4, https://links.lww.com/EANDH/B690), which both showed the same pattern of effects. However, that may not always be the case in other datasets, and the fact that differences could be observed here in both accuracy and response time highlights that neither measure alone captured the full impact of having a CI or the differences between presentation modes. In this respect, the listening efficiency metric offers a more complete picture of the true nature of between- and within-group effects. Though it remains to be proven by further study, we believe that the listening efficiency approach holds promise as a sensitive outcome measure in other situations where accuracy may be close to ceiling level and effects potentially subtle, for example, when evaluating different hearing-device processing algorithms.

### Limitations

Participants were recruited into the present online study via e-mail and social media. This could have introduced selection bias since the people we reached may already have been more familiar with online technology and thus with online communication. Unfortunately, we captured limited information about participants’ frequency of video-calling activity and familiarity with the use of real-time captioning to support online communication specifically. Despite the two groups of participants being approximately age-matched, a greater number of people with a CI were retired compared with their AH peers (62% and 30% retirees, respectively). Such imbalance might have contributed in part to the observed difference in performance between groups in the speech-recognition task, based on the assumption that individuals still in the workforce may, on average, be more frequent users of online communication tools than retirees. We also cannot exclude the possibility of subclinical differences in cognitive health/ability between the two groups, which could have impacted the listening efficiency results independently of, or in interaction with, the difference in hearing status.

Another limitation of the study, related to the online context, is that we had limited control over sound reproduction settings and user input devices. To reduce any impact this may have had, participants received detailed set up instructions and were encouraged not to adjust settings or devices once the experiment had commenced, providing a degree of within-subject (across presentation modes), though not between-subject, control. Given that our LBA-based analysis method seeks to model response time distributions, differences in input device could be a potential between-subject confound (Warburton et al. 2024). However, we note that the difference in response time seen between the CI and AH groups, which averaged around 600 msec (Supplemental Digital Content 4, https://links.lww.com/EANDH/B690), was an order of magnitude larger than the reported difference in response time for touch versus mouse inputs (touch input being ~65 msec faster on average) (Sauter 2024). This, together with the fact that estimation of a “non-decision time” parameter within the LBA model provides some robustness against differences in the speed of motor execution, leads us to conclude that a relatively minor difference in the distribution of mouse versus touchscreen input devices between our CI and AH groups is unlikely to have had any appreciable impact on the reported outcomes.

A further limitation of the present study concerns the use of prerecorded stimuli to simulate online communication, which would ordinarily be dynamic and interactive. While the conversations used during the preference task were real prerecorded online conversations, the sentence-length video clips used for the speech-recognition task were further removed from a typical online conversation. Nonetheless, the BBC-LRS2 clips include high-context sentences covering a wide range of topics, with different speakers, and from different locations, which may have to some degree represented the variety in talkers, backgrounds, and camera angles experienced in online video calls. Regarding the generalizability of our findings, another important consideration is the representativeness of the captions in our stimuli. We sought a reasonably consistent captioning style across the preference and speech-recognition tasks, that, in the opinion of the authors, was broadly representative of the type of captioning experienced in popular web-based video-calling platforms at the time the experiment was conducted in 2021/22. Nonetheless, previous research indicates that numerous factors can affect the perceived quality and utility of captions, including their accuracy (Kafle & Huenerfauth 2019), visual appearance (Berke et al. 2019), timing (Sandford 2015), and placement (Amin et al. 2021). Variation in these factors was not well captured by the fixed stimuli used in the present experiment, and it must be noted that the quality of automatic live captioning systems is likely to continue to improve with advances in research and technology. One important point that can be made is that, because the captions used in the speech-recognition task were generated from accurate sentence transcriptions, captioning inaccuracy cannot have played a role in limiting the amount of benefit that was observed in terms of listening efficiency; however, other aspects of caption presentation may have done.

## CONCLUSION

We provided evidence that adults with a CI generally prefer an “audio-visual plus captions” presentation mode during online communication, and that they benefit objectively from access to lipreading and captions in terms of being able to more quickly arrive at an accurate perception of what was said. However, participants reported some perceived drawbacks of captions, including missing some non-verbal information from the video stream and a cognitive cost associated with the need to rapidly switch attention back and forth between the audio-visual and textual streams. Objectively, even with access to video and accurate captions, people with a CI were still, on average, less efficient listeners than their AH peers. Together, these results indicate that, while captions are valued by, and beneficial to, people with a CI, they do not fully alleviate the challenges that this group faces with online communication. Our participants made several suggestions for ways in which online captioning systems could potentially be improved, some of which are already the subject of active research.

## ACKNOWLEDGMENTS

This research was jointly funded by the Royal National Institute for Deaf People (RNID) and Advanced Bionics through a PhD studentship (grant reference: S53, awarded to F.P.P.), and supported by the NIHR Nottingham Biomedical Research Centre (funding reference number BRC-1215-20003). The funders had no role in study design, data collection, analysis, decision to publish, or preparation of the manuscript. The views expressed in this article are those of the author(s) and do not necessarily represent those of the NHS, the NIHR, or any affiliated organizations.

## Supplementary Material

## Footnotes

* In a centered parameterization, sampling is performed over a parameter directly at its original location and scale. For example, in the case of a parameter *β* that is normally distributed with mean *μ* and SD *σ*, we might sample over *β* ~ N(*μ*, *σ*^2^). However, in the context of fitting a hierarchical Bayesian model, this can result in poor sampling behavior and possibly failure to converge (bearing in mind that we are likely to be estimating the values of *μ* and *σ* at the same time as estimating *β).* Sampling behavior can often be improved by switching to a noncentered parameterization, in which we sample over a new parameter *β*_raw_ that follows a standard normal distribution, *β*_raw_ ~ N(0, 1), and then transform back to the original location and scale using *β = μ + σ × β*_raw_.

† Response caution is calculated as *b* − *A*/2, where *b* is the response threshold (amount of evidence needed to trigger a response) and *A* is the maximum amount of starting evidence at the beginning of a trial. It is a measure of how much evidence an individual waits to accumulate, on average, before initiating their response, and reflects that individual’s approach to the speed-accuracy trade-off.
