# Supplementary material for: Preferences and Listening Efficiency of Adults With Cochlear Implants During Online Communication
Source: Ear Hear. 2025 Sep 4;46(6):1661–73. doi: 10.1097/AUD.0000000000001702 (PMC12533766; doi:10.1097/AUD.0000000000001702)
Supplement: Supplementary file 1 [file aud-46-1661-s001.pdf]

SUPPLEMENTAL DIGITAL CONTENT 1: QUESTIONNAIRE ITEMS

1.- Demographic and hearing questions

Demographic Information

Q1. How old are you? Please, enter your age in years into the box below:

Q2. Which gender best defines you?

- *Female*
- *Male*
- *Other*
- *Prefer not to answer*

Q3. What is your current employment status?

- *Homemaker*
- *Employed full time*
- *Employed part time*
- *Self-employed*
- *Student*
- *Unable to work*
- *Retired*
- *Furloughed or unable to work due to COVID-19*
- *Unemployed*
- *Unsure*
- *Other*
- *Prefer not to say*

Hearing Information

The following questions are about your hearing in the left ear.

Q4. How is your hearing in your left ear? *If you are unsure, please select the option that you feel best describes your hearing in this ear.*

- *Good- no loss*
- *mild loss*
- *moderate loss*
- *severe loss*
- *profound loss*

Q5. Do you use a hearing device in your left ear?

- *None*
- *hearing aid*
- *cochlear implant*
- *other*

If cochlear implant selected:

Q5.1. For how long have you been using your cochlear- implant in the left ear? Please enter a number in years into the box below. *Enter 0 if you have less than one year experience with your implant.*

Q5.2. Do you know roughly how old you were when you lost your hearing in your left ear? *Please indicate the option that applies according to the age at which you lost your hearing.*

- *Infant (0-1 year)*
- *Toddler (1-3 years)*
- *Pre-schooler (3-5 years)*
- *Childhood (6-12 years)*
- *Teenager (13-19 years)*
- *Twenties*
- *Thirties*
- *Forties*
- *Fifties*
- *Sixties*
- *Seventies or over*
- *I don’t know*

If hearing aid selected:

Q5.1. Do you use your hearing aid regularly on a daily basis?

- *Yes*
- *No*

Q5.2. Are you using the hearing aid right now (during the experiment)?

- *Yes*
- *No*

If other device selected:

Q5.1. If you selected other please write the name of the device you use in your left ear in the box below:

The following questions are about your hearing in the right ear.

Q6. How is your hearing in your right ear? *If you are unsure, please select the option that you feel best describes your hearing in this ear.*

- *Good- no loss*
- *mild loss*
- *moderate loss*
- *severe loss*
- *profound loss*

Q7. Do you use a hearing device in your right ear?

- *None*
- *hearing aid*
- *cochlear implant*
- *other*

If cochlear implant selected:

Q7.1. For how long have you been using your cochlear- implant in the right ear? Please enter a number in years into the box below. *Enter 0 if you have less than one year experience with your implant.*

Q7.2. Do you know roughly how old you were when you lost your hearing in your right ear? *Please indicate the option that applies according to the age at which you lost your hearing.*

- *Infant (0-1 year)*
- *Toddler (1-3 years)*
- *Pre-schooler (3-5 years)*
- *Childhood (6-12 years)*
- *Teenager (13-19 years)*
- *Twenties*
- *Thirties*

- *Forties*
- *Fifties*
- *Sixties*
- *Seventies or over*
- *I don't know*

If hearing aid selected:

Q7.1. Do you use your hearing aid regularly on a daily basis?

- *Yes*
- *No*

Q7.2. Are you using the hearing aid right now (during the experiment)?

- *Yes*
- *No*

If other device selected:

Q7.1. If you selected other please write the name of the device you use in your right ear in the box below:

Ways of communication

Q8. In everyday life, to what extent do you rely on these ways of communication?

Please provide an answer to each way of communication mentioned below.

	Never	Rarely	Sometimes	Often	Almost Always
Listening					
Lipreading					
Facial expressions					
Sign Language					
Text transcriptions					

2.- Setup instructions

Getting set up

Please make sure you wear your hearing devices e.g., cochlear implant(s) or hearing aid(s) as you would normally do. Once adjusted, please don´t do any further adjustment during the experiment.

This study requires you to watch and listen to video and audio clips. You can use either your computer/tablet loudspeakers or headphones. Once you decide how you will listen, please use the same setup throughout the experiment.

Next button

Q9. Which sound reproduction setting are you using during the experiment? *Please use the set up that you would normally use during a video call or the one you feel more comfortable with.*

- *Loudspeakers from computer or tablet*
- *Headphones*
- *Stream sounds to your hearing devices*
- *Other*

Next button

Sound check

1.Click the play button below to hear an audio example.

2.Adjust the volume on your device so that the sound is at a comfortable level (not too quiet, not too loud). You can replay it as many times as you like.

If you didn´t hear anything, ensure your sound is not muted and that the volume is turned up sufficiently, then press play again.

Play button

☐ By clicking the box I confirm that the sound is at a comfortable level.

Button Selection

After listening to the audio and video clips, you will be required to complete a task by selecting some buttons. If you have a touchscreen computer, you can tap the buttons with your finger. Otherwise, you can use your mouse to click the buttons. Whatever option you decide, please use the same approach throughout the experiment.

Q10. I confirm that I am using a:

- *Touchscreen device (e.g., tablet)*
- *Computer with mouse*

Next button

Great! Now you are ready to start the test!

Please, do not make any changes to your device settings, change the volume or switch to any other apps until the experiment is complete.

If you are ready to start, click 'Continue'

To go through the instructions again, click 'Back'

Back button ***Continue button***
