# Supplementary material for: Preferences and Listening Efficiency of Adults With Cochlear Implants During Online Communication
Source: Ear Hear. 2025 Sep 4;46(6):1661–73. doi: 10.1097/AUD.0000000000001702 (PMC12533766; doi:10.1097/AUD.0000000000001702)
Supplement: Supplementary file 2 [file aud-46-1661-s002.pdf]

SUPPLEMENTAL DIGITAL CONTENT 4: SEPARATE ANALYSES OF ACCURACY AND RESPONSE TIME

The following analyses were performed in R using the 'BRMS' package for Bayesian statistics.

In both models (accuracy and response time), broad uninformative priors were used, and four independent Markov chain Monte Carlo (MCMC) chains were run with 8000 iterations each (2000 warm-up iterations, 6000 sampling iterations). Successful model convergence was verified by confirming that all R-hat diagnostic values were ≤1.01 (R-hat = 1 at full convergence).

Individual differences were accounted for by including a random effect of "Participant" (intercept only) in each model, with the hyper-parameter (standard deviation) of the random effect allowed to vary between the cochlear-implant (CI) and acoustic-hearing (AH) groups.

Accuracy

Trial-level accuracy (count of keywords correctly identified, out of three per trial) was modelled using a beta-binomial distribution. The model included fixed effects of Group (CI/AH) and Presentation Mode (Audio/Video/Captions), with the following formula:

```
KeywordsCorrect | trials(KeywordsTotal) ~ 0 + Group:PresentationMode + (1|gr(ParticipantID, by = Group))
```

Posterior predictive checks confirmed that the model gave a good fit to the data.

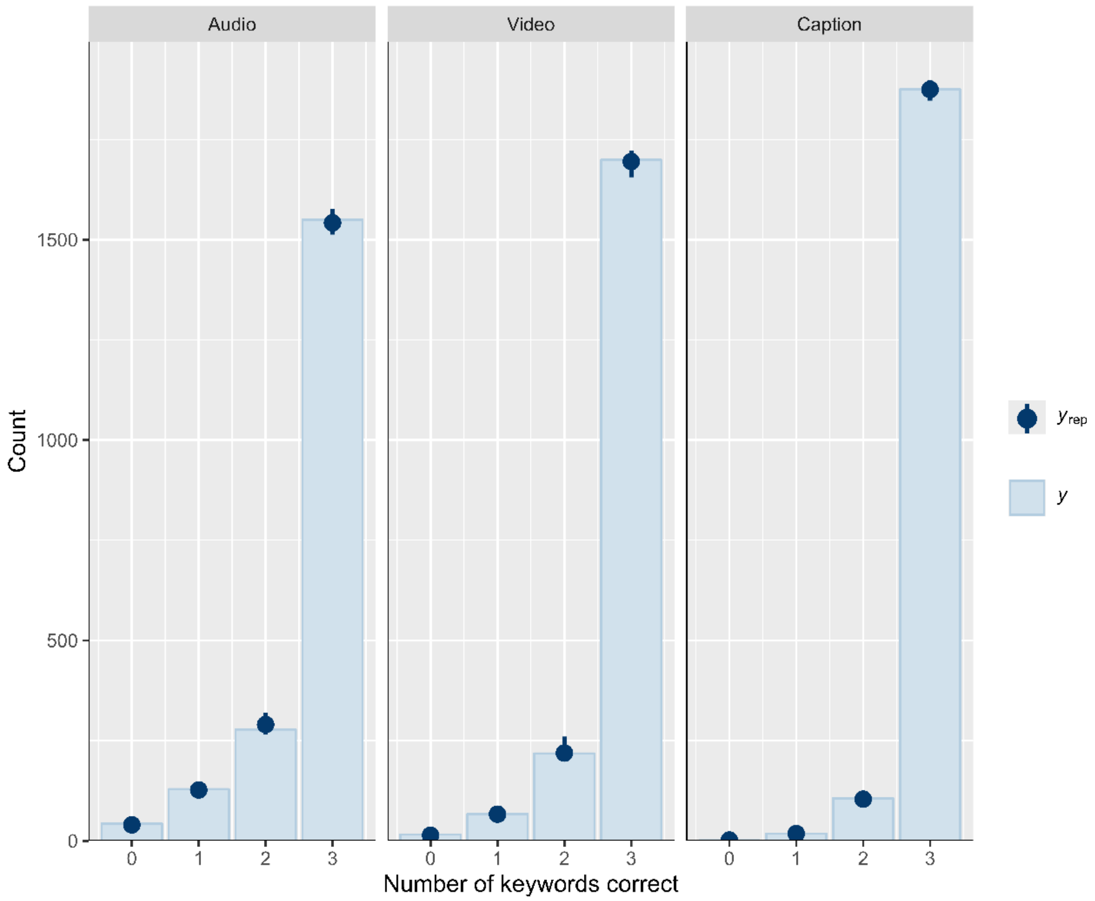

Figure: Posterior predictive checks for the accuracy model (light blue bars = raw data; dark blue point intervals = model predictions over 100 draws)

Expected percentage correct scores were calculated from the model posterior for each combination of Group and Presentation Mode:

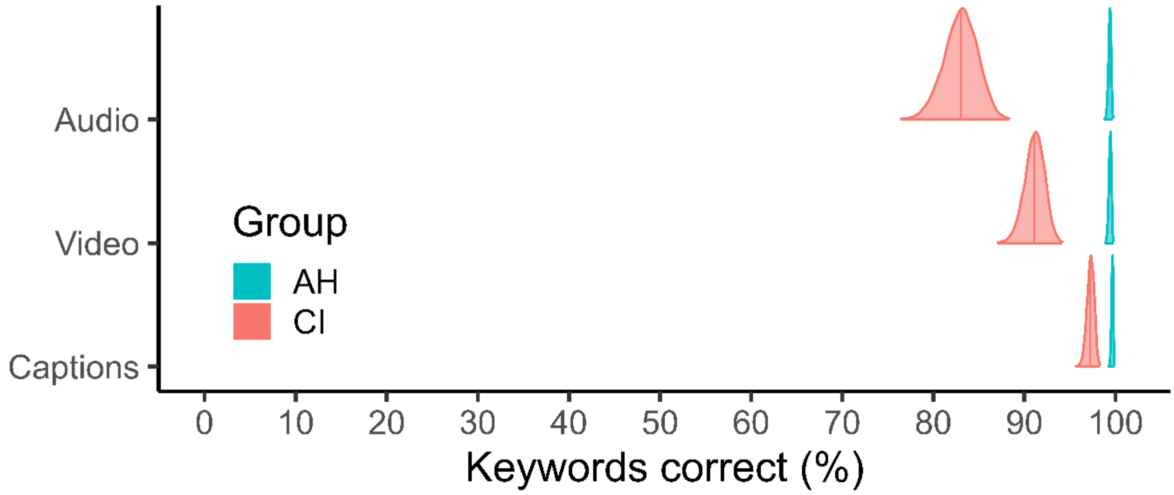

Figure: Expected values of accuracy (percentage keywords correct) from the model posterior (solid vertical lines represent median values and the distributions extend to cover the central 99% probability mass of the posterior)

Accuracy for the AH group was effectively at ceiling (~100% correct), regardless of presentation mode, with a high level of certainty (narrow posterior distributions).

For the CI group, median expected accuracy was 83% for audio-only presentation, 91% for audio-visual presentation, and 97% for audio-visual presentation with captions. The differences in accuracy between presentation modes (within the CI group) were highly robust: credibility of greater accuracy for audio-visual than for audio-only presentation 24000/24000 ≈ 100%; credibility of greater accuracy for audio-visual presentation with captions than for audio-visual presentation without captions 24000/24000 ≈ 100%.

Response time

For ease of interpretation, response times were analysed for correctly identified keywords only (i.e., error responses were excluded).

Response times were modelled using a shifted log-normal distribution. The model included fixed effects of Group (CI/AH), Presentation Mode (Audio/Video/Captions) and Keyword Position (1–3), with the following formula:

```
rt ~ 0 + Group:PresentationMode + KeywordPosition + (1|gr(ParticipantID, by = Group))
```

Keyword Position was included to account for the fact that correct responses tended to be quicker for keywords appearing later in the test sentences.

Posterior predictive checks confirmed that the model gave a reasonable fit to the data.

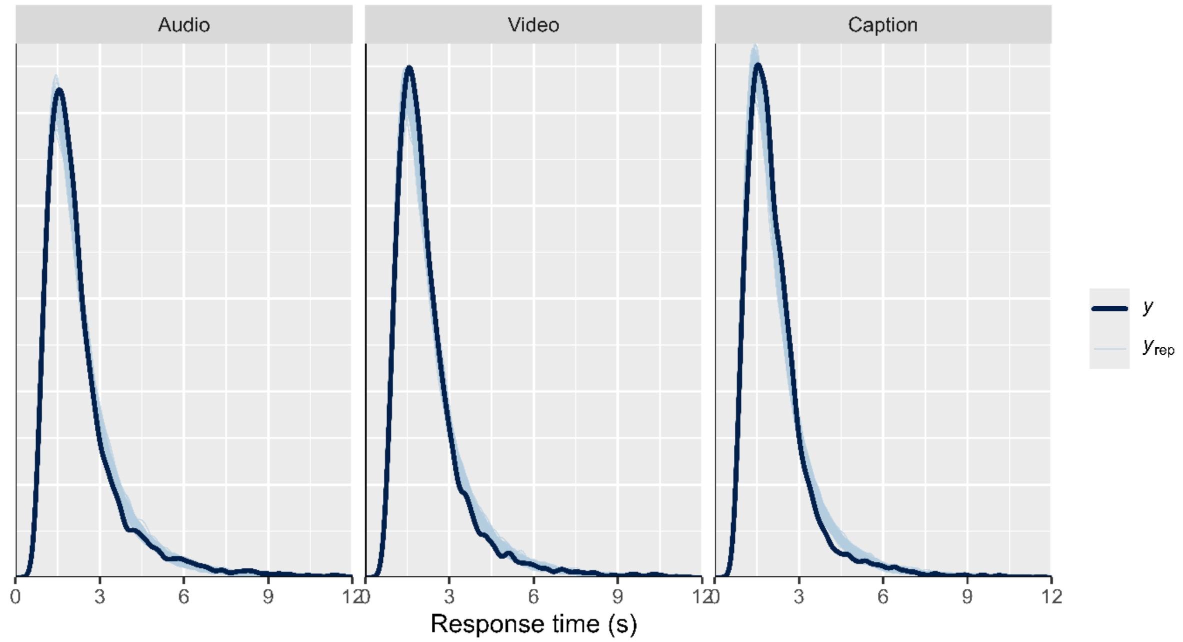

Figure: Posterior predictive checks for the response-time model (dark blue line = raw data; light blue lines = model predictions over 100 draws)

Expected median response times for correct trials (averaged across keyword positions) were calculated from the model posterior for each combination of Group and Presentation Mode:

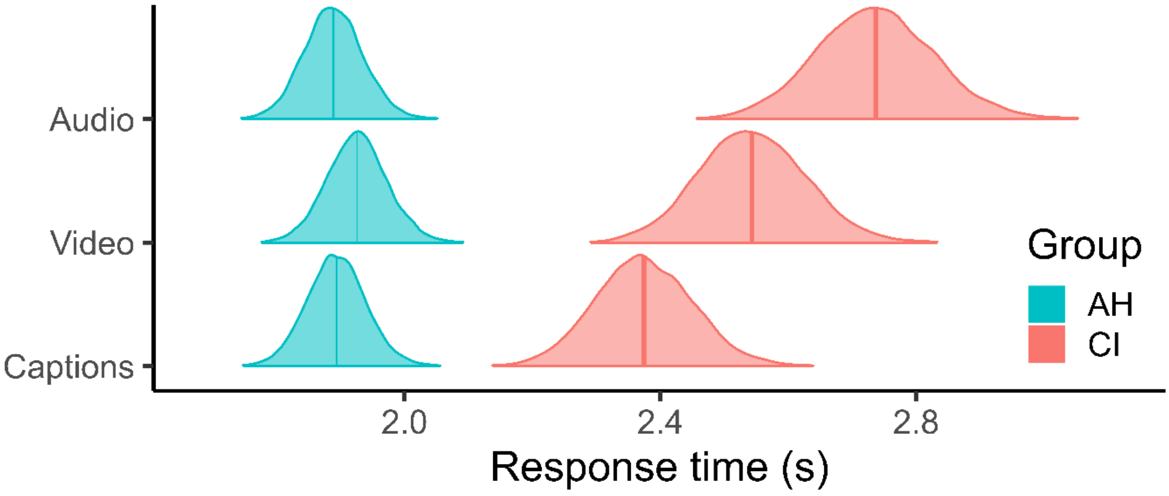

Figure: Expected values of response time for correct trials (averaged across keyword positions) from the model posterior (solid vertical lines represent median values and the distributions extend to cover the central 99% probability mass of the posterior)

For the AH group, expected response times were consistent across presentation modes, with a median at around 1.9 s and strongly overlapping posterior distributions.

For the CI group, median expected response times were longer than for the AH group, and more dependent on presentation mode: 2.7 s for audio-only presentation, 2.5 s for audio-visual presentation, and 2.4 s for audio-visual presentation with captions. The differences in expected response time between presentation modes (within the CI group) were highly robust: credibility of shorter response time for audio-visual than for audio-only presentation 24000/24000 ≈ 100%; credibility of shorter response time for audio-visual presentation with captions than for audio-visual presentation without captions 24000/24000 ≈ 100%.
