# Supplementary material for: Preferences and Listening Efficiency of Adults With Cochlear Implants During Online Communication
Source: Ear Hear. 2025 Sep 4;46(6):1661–73. doi: 10.1097/AUD.0000000000001702 (PMC12533766; doi:10.1097/AUD.0000000000001702)
Supplement: Supplementary file 3 [file aud-46-1661-s003.pdf]

SUPPLEMENTAL DIGITAL CONTENT 2: THEMES AND CATEGORIES IN RESPONSES TO FREE-TEXT QUESTIONS

2.1 COCHLEAR IMPLANT GROUP

Conversation A

Table 2. 1. *Themes, categories, number of mentions (N.), and example statements given by CI users in Conversation A, in response to questions: "if this was a real video call that you were involved in, which mode would you prefer to use?", "please tell us why this was your preferred mode", "were there any downsides to your preferred mode?". The number in brackets beside each presentation mode indicates the number of participants who selected that particular mode.*

Mode	Theme	Category	N.	Example
**Audio (1)**	Reason	Difficult lipreading	1	"I preferred the audio, as with the video I found the males lips hard to read"
	Downside	Accent	1	"The accent"
**Video (3)**	Reason	Lipreading	2	"I could read their lips which aided understanding whereas audio only I found the participants speech a little fast to follow at first"
		Facial expressions	1	"See faces"
		Caption distracting/inaccurate	1	"I prefer it to the mode with the closed captions as this is sometimes distracting as they load one word at a time. Furthermore, the captions are often slightly inaccurate and this can make my understanding worse as I am receiving inputs that don't match"
	Downside	Connection quality	1	"Relying on video depends on the internet connection. If the connection is bad the video can be blurry which makes it hard to lipread. A bad connection can also distort the sound"
		Video quality/camera position	1	"Shadow on the ladies face and sitting to the side slightly"
		Caption needed depending on conversation type	1	"if a technical conversation or background noise would use captioning too"
**Captions (46)**	Reason	Lipreading not possible (unfamiliar accents/unclear speech, audio-video not in sync)	17	"The man was quite difficult to follow, even with lip reading. He didn't move his mouth in a natural manner"
		Allows to understand all conversation/ less effortful	15	"With the captions I could understand all the conversation.", "Captions - I found it was less effort to understand the conversation."
		Provides confirmation/confidence	10	"the captions help to confirm my understanding and clarify any missed words", "It gave me confidence".
		Accurate/ in-sync transcriptions	3	"The text seemed very accurate although it didn't filter out the speakers' hesitations and repetitions."," The captions were in sinc with the speakers in this clip"
		Useful when poor sound quality/noisy background	3	"there was some interference, a whistling sound that detracted from the audio"
		Useful when poor video quality/ obscured faces	3	"captions essential as video quality not good","The female participan't face was partly shadowed. When faces are obscured or blurred on a video call it makes things that bit harder"
		Useful when Unfamiliar topic/ difficult conversation	3	"The context was a lot harder to grasp - maybe that made it harder too"
		Useful when overlapping speech	2	"they talked over each other… so captions were helpful"

		Useful for fast speech	1	“The speed of the talking and lack of lipmovement from the participants left me baffled”
	Downside	No downsides (if accurate, readable, and in sync with speech)	21	“No, I followed it okay”, “No, as long as the captions are big enough to read easily and keep pace with the dialogue.”
		not accurate/delayed	6	“captions was slow to catch up”, “if it is automatic captioning, it often is wrong in accuracy”.
		Need to concentrate on text and miss people’s faces	5	“I miss some of the interaction by looking down at the captions”
		Cognitive overload when looking at captions and people at the same time	3	“Cognitive overload as usual... if I'm looking at the text then I'm not looking at the faces. Perhaps, in a more ideal world, each person's speech would appear in speech bubbles besides their heads... like a comic book”
		Absent during overlapping speech	2	“Lots of talking over each other in that video so no captions at times”
		Subtitle position	1	“The positioning of the subtitles, it would be useful to have some control over that”
		Difficulty to know whom the caption refer to	1	“One is not always sure to whom the captions refer”

Conversation B

Table 2. 2. *Themes, categories, number of mentions (N.), and example statements given by CI users in Conversation B, in response to questions: "if this was a real video call that you were involved in, which mode would you prefer to use?", "please tell us why this was your preferred mode", "were there any downsides to your preferred mode?". The number in brackets beside each presentation mode indicates the number of participants who selected that particular mode.*

Mode	Theme	Category	N.	Example
**Audio (2)**	Reason	Clear speech/Easy topic	2	"Participants speech was clear and topic easily followed so didn't need video"
	Downside	None	2	"None at all"
**Video (13)**	Reason	Clear speech	7	"very clearly enunciated and easily followed"
		Facial expressions/body language	6	"I like to see the faces and expressions"
		Easy to lipread	4	"Video mode allowed me to lip read"
		Captions distracting (out of sync/delayed)	3	"I found audio only hard to hear and video with captions distracting from speech and hard to follow."
	Downside	None	10	"Not really"
		Video and audio out of sync	1	"The delay in video and speech was distracting"
		Concentration required	1	"Requires concentration to follow the conversation"
		No captions as back-up	1	"If I missed the odd word there was no way of checking (i.e. no captions)"
**Captions (35)**	Reason	Allows to understand all conversation/less effortful	17	"made it so much easier to understand with the captions and less effort involved with straining to lipread and make use of residual hearing"
		Provides confirmation/ back-up/confidence	12	"I barely looked at the captions, but it was good to have them there. To quickly cast my eye down to see what that missed word was", " I can relax more knowing that the captions are there so that I don't miss out."
		Useful when audio and video out of sync	2	"because the talking was delayed in what was being said"
		Useful for fast speech/volume variations	2	"because the speak too fast for me and their pitch is too high"
		Useful when lipreading is difficult (accents)	2	"I found the female with the glasses hard to lip read"
	Downside	None	19	"Not really. it would be fine to participate in a real conversation in this mode."
		Miss people's faces	3	"by looking down at the captions I miss a bit of the visual interaction and facial expressions"
		Cognitive overload	1	"Cognitive overload... particularly with trying to synchronise the speech and captions in my head. In everyday life, I would only look at the text when I'm unsure of what's been said."
		Captions out of sync (Delayed)	4	"Captions don't always sinc with lip patterns"
		not accurate	1	"auto palantypist doesn't always get it right"
		Lack of punctuation	1	"Punctuation the captions would improve things..."
		Difficulty to know whom the caption refer to	2	"It would be better if different peoples conversations were in different coloured text, as its difficult to understand who is saying what!"
		Overlapping talk	2	"people tend to talk across each other"
		Captions position	1	"Yes, the biggest problem is where the subtitles are situated on the screen. They are usually down the bottom of the screen which makes it difficult to interact with the individual, I would prefer them to be mid screen"

Conversation C

Table 2. 3. *Themes, categories, number of mentions, and example statements given by CI users in Conversation C, in response to questions: "if this was a real video call that you were involved in, which mode would you prefer to use?", "please tell us why this was your preferred mode", "were there any downsides to your preferred mode?". The number in brackets beside each presentation mode indicates the number of participants who selected that particular mode.*

Mode	Theme	Category	N.	Example
**Video (19)**	Reason	Clear speech/good sound quality	10	"Both speakers were easy to understand and I had no problem following the conversation. Even when the captions were showing, I didn't need use them"
		Easy to lipread (video and audio in sync)	8	"the lip-sync was accurate so the combination of lip-reading and sound made it easy to understand"
		Captions out of sync	7	"I would normally want the captions, but they are out of sync with the speaker, so I have gone for the video."
		Seeing the speakers improves the conversation experience (facial expressions/body language)	5	"I was able to understand both people very easily and clearly but the video added to the experience by showing their facial expressions and obvious enjoyment of the conversation. I did not need to use subtitles."
		Makes conversation more personal	1	"Just more personal to have a video call"
	Downside	None	10	"None; in this case the video was good and the voices and lips were synchronised"
		No captions	6	"I can't check from the captions when I'm not certain what was said."
		Video and audio out of sync	2	"In some parts there was a lag between the video & audio that could have made lipreading difficult"
		Concentration required	1	"I have to concentrate to understand but I am still able to follow the conversation well"
**Captions (31)**	Reason	Provides confirmation/back up	13	"It helps to be able to double check what I thought I heard was correct"
		Allows to understand all conversation/less effortful	13	"I could follow every word with the video with captions without concentration", "Those speaking in the videos, were speaking exceptionally clearly in ideal conditions, with not background noises. Watching speakers, lipreading plus captioning is the best accessibility practice."
		Useful when lipreading is difficult (accents, distance from camera, fast speech)	4	"the pattern of the lips was sometimes difficult to follow", "It's really hard work to concentrate to lipread people 'further away' than if it were a face to face conversation. Also people speaking too fast and I cannot ask them to slow down a bit"
		Accustomed to watching TV with subtitles	2	"I have become used to watching the television with subtitles in fact if there was a programme I wanted to watch and there were no subtitles I tend to abandon it."
	Downside	None	19	"Not as long as the captions are easy to see and keep pace with the dialogue."
		Not in sync	5	"The syncing of text to voice is a little off, so there's a feeling of cognitive overload"
		Not accurate	3	"some of the words were wrong"
		Miss people's faces	3	"With captions I could not look at the faces but that is a small price to pay"
		Requires ability to read quickly	1	"One has to read very quickly."

2.2 NORMAL HEARING GROUP

Conversation A

Table 2. 4. *Themes, categories, number of mentions (N.), and example statements given by NH participants in Conversation A, in response to questions: "if this was a real video call that you were involved in, which mode would you prefer to use?", "please tell us why this was your preferred mode", "were there any downsides to your preferred mode?". The number in brackets beside each presentation mode indicates the number of participants who selected that particular mode.*

Mode	Theme	Category	N.	Example
**Audio (6)**	Reason	Clear/ Easier to follow	3	"The dialogue is clear and I am able to focus on the conversation. If the audio was unclear I would opt for visual and then text , if really bad"
		No distraction from video or captions	3	"The images were a little distracting as one of the presenters was not sitting straight in front of the screen"
	Downside	None	4	"No"
		No facial expressions	1	"If I were talking to family or business I would then prefer the visual to see facial expressions"
		Long pauses	1	"There were quite long pauses so potentially you could be wondering if the connection was lost without visual clues"
**Video (32)**	Reason	Facial expressions/see who is talking	16	"could put a face to the voice and know which of them was talking"
		Easier to understand (if poor audio quality or overlapping talk)	8	"Could understand better what was being said especially when they talked at same time"
		Caption distracting/inaccurate	7	"Captions were sometimes inaccurate and generally distracting."
		Feels more natural/ better engaged/inclusive	6	"Again, it felt natural. I felt better engaged.", "more like F2F encounters".
		More common in daily meetings	2	"I use this mode in daily work life"
	Downside	None	19	"None in this mode"
		Video can be distracting	4	"Shadow on one person slightly annoying", "Only the distraction of unfamiliar backgrounds"
		Difficult to understand when overlapping talk	3	"Still difficult to understand when both participants were speaking but captions did not resolve that problem either in this example."
		Captions useful for accents, unfamiliar or unclear speech	3	"I would certainly appreciate captions if a person's accent or dialect was less familiar and/or difficult for me to understand quickly"
		Connection quality/background noise	2	"Slight buzz noise in background "
		Cognitive load	1	"Video calls are more tiring than audio calls"
**Captions (12)**	Reason	Useful when poor sound quality, noisy background or unclear speech	7	"There was a buzzing sound in the background which was a little off putting and the audio had a slight echo in the video option, so I preferred to read the captions in this instance for extra reassurance"
		Useful when overlapping talk	4	"At some points the speakers spoke at the same time and it was difficult to make out what they were saying so it was useful to have the captions."
		Easier to understand/more clarity	3	"I can see who is talking and clarity on the words spoken, if the transcription is accurate"
	Downside	Miss visual information/facial expressions	5	"I miss some of the interaction by looking down at the captions"

		Not accurate	4	“Inaccuracies in auto subtitling”
		None	3	“No, I could see the participants, hear them and read the transcript as a backup.”
		Difficult to read due to lack of punctuation and scrolling text	2	“no punctuation and the transcript disappears after a couple of lines”

Conversation B

***Table 2. 5**. Themes, categories, number of mentions (N.), and example statements given by NH participants in Conversation B, in response to questions: "if this was a real video call that you were involved in, which mode would you prefer to use?", "please tell us why this was your preferred mode", "were there any downsides to your preferred mode?". The number in brackets beside each presentation mode indicates the number of participants who selected that particular mode.*

Mode	Theme	Category	N.	Example
**Audio (4)**	Reason	Clear speech	2	"The sound and voices were very clear and there was no need to see any of the presenters on a screen to keep up with the conversation"
		Only one input to concentrate on	2	"With audio there was only one thing to concentrate on"
	Downside	None	2	"None at all"
		Requires good hearing and concentration	2	"Relies on good hearing and concentration"
		Lose the personal interaction	1	"Lose the personal touch and ability to see facial expression of those speaking"
**Video (40)**	Reason	Facial expressions enhance comprehension (who speaks, their attitude, the tone of the conversation)	29	"I can see who is speaking as well their facial expressions which helps to understand the tone of the conversation", "Prefer to see facial expression as this adds meaning to the words"
		It's more pleasant, more personal and feels more involved and natural	11	"felt more involved, more natural, real people", "felt more like a normal face to face conversation."
		Captions distracting or out of sync	7	"Captions were distracting and unnecessary", "captions delayed"
		Lipreading	3	"Partial lipreading"," "I also use lip-reading even if people speak clearly and I do not have hearing loss"
	Downside	None	29	"Not that I can think of"
		Video can be distracting	5	"Possibly could get distracted by things that could be seen other than conversation"
		Captions needed for missing words (especially when accents, unfamiliar speech or technical discussion)	5	"no, but if someone had a strong accent or English was not their first language, I might also read the live transcription"
		Video and audio out of sync	2	"Audio-Visual lag"
		Poor video quality	1	"Quality of picture"
**Captions (6)**	Reason	Easier to understand/more clarity	5	"More clarity", "Sometimes you can't make out exactly what a person said and the captions are a good fail safe."
		Useful when poor connection signal	1	"Best proof against disturbances in the signal"
	Downside	None	5	"No. Text helped supplement"
		Miss visual information	1	"You might miss some visual information while looking at the scrolling text."

Conversation C

Table 2. 6. *Themes, categories, number of mentions (N.), and example statements given by NH participants in Conversation C, in response to questions: "if this was a real video call that you were involved in, which mode would you prefer to use?", "please tell us why this was your preferred mode", "were there any downsides to your preferred mode?". The number in brackets beside each presentation mode indicates the number of participants who selected that particular mode.*

Mode	Theme	Category	N.	Example
**Audio (5)**	Reason	Clear speech	2	"clear speakers, no interruptions, so concentrated on content"
		Audio and video out of sync	3	"The sound wasn't quite in sync with the video which I found a little off putting. The text caption wasn't fully synched either so I preferred to just listen to this one"
	Downside	None	2	"No, I can listen and do other tasks at the same time"
		Miss facial expressions	3	"I missed seeing the lively expressive faces"
**Video (36)**	Reason	Facial expressions enhance comprehension (who speaks, their attitude, the tone of the conversation)	22	"I wanted to see the expressions on their faces", "I can observe expressions and see that someone is not distracted or disengaged"
		Captions distracting (not accurate, lack of capitalization, out of sync)	12	"I do find having text can be distracting", "I found the transcription irritating as there were lower case letters where there should have been capitals (I am a grammar geek!)"
		It's more pleasant, more personal and feels more engaging and natural	10	"I like to see the people I am speaking to, particularly as we've not had much opportunity for interaction for 2 years."
		Lipreading	3	"I also use lip-reading even if people speak clearly and I do not have hearing loss"
		Common mode in daily meetings	2	"It's my usual way of holding a meeting over the internet"
	Downside	None	22	"None I can think of"
		Captions useful if missing words (especially when accents, unclear or fast speech)	7	"Delivery a mixture of slow and fast sections of speech so text may assist understanding"
		Video and audio out of sync	3	"At some points in the video there was a mismatch between the video and the sound"
		Video can be distracting	3	"some backgrounds can be distracting"
		Be on camera	1	"I have to be on camera as well which means I have to be careful with my reactions."
**Captions (9)**	Reason	Easier to understand/more clarity (especially when accents, unclear or fast speech)	6	"it helped to understand better what they were saying.", "Sometimes you may mishear or misinterpret what was said because of regional accents"
		Useful when poor connection signal (audio and video out of sync)	3	"It was easier to follow the conversation as the sound and vision wasn't always in sync'"
		Help to stay focus/remember the conversation	2	"They were speaking quite slowly so it was hard to stay focussed. The captions to read made it more interesting"
	Downside	None	4	"No"
		Miss facial expressions	3	"Couldn't really read the captions and see the expressions at the same time"
		Not accurate	2	"Just the questionable reliability of auto subtitling."
