# Supplementary material for: Preferences and Listening Efficiency of Adults With Cochlear Implants During Online Communication
Source: Ear Hear. 2025 Sep 4;46(6):1661–73. doi: 10.1097/AUD.0000000000001702 (PMC12533766; doi:10.1097/AUD.0000000000001702)
Supplement: Supplementary file 4 [file aud-46-1661-s004.pdf]

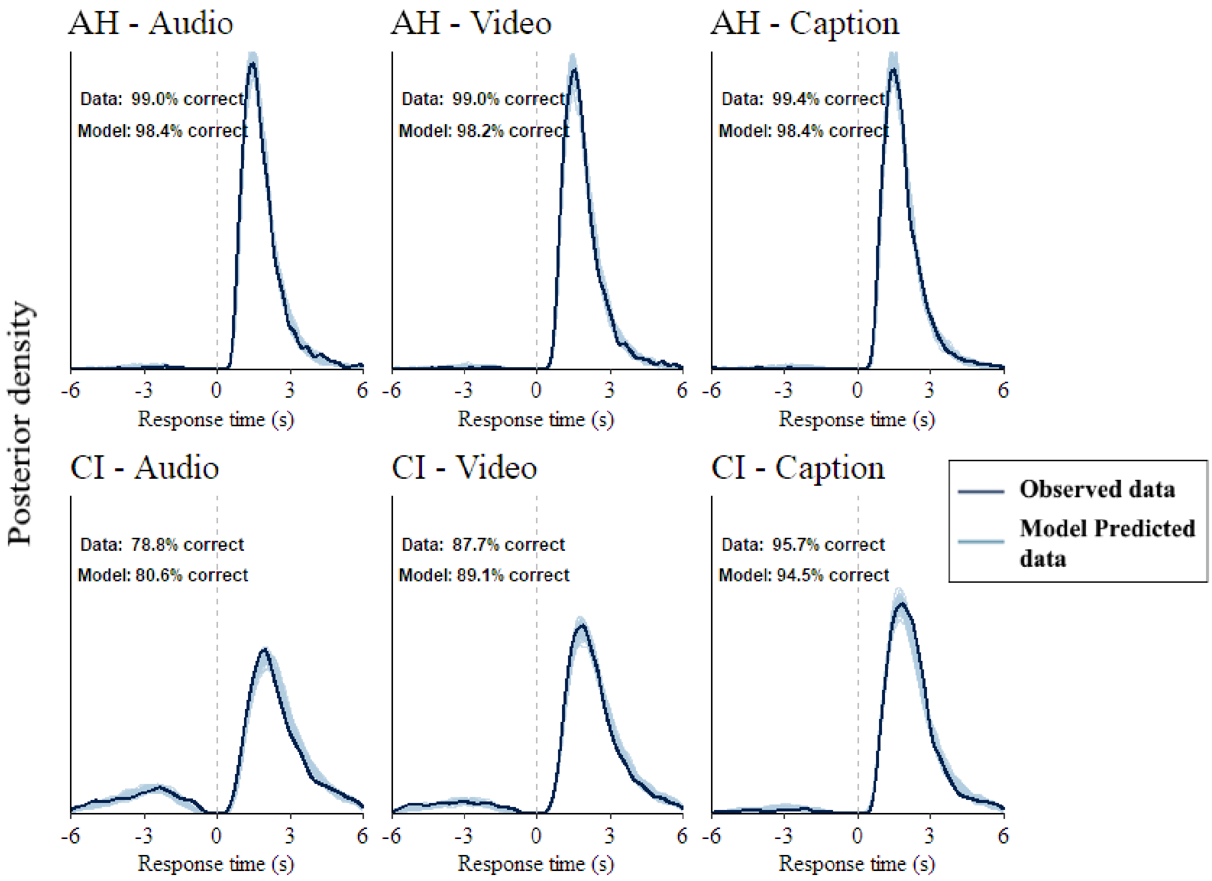

Supplemental Digital Content 3. *Posterior predictive checks for each group (AH and CI groups shown in the upper and lower rows, respectively) and presentation mode (audio, video, and captions modes shown from left to right across columns). Response times for incorrect responses are plotted as negative values. Solid dark lines represent the observed data and light blue lines represent the model predicted data (first 50 posterior draws).*
